# Comparative genomics reveals that loss of lunatic fringe (*LFNG*) promotes melanoma metastasis

**DOI:** 10.1002/1878-0261.12161

**Published:** 2018-01-07

**Authors:** Martin Del Castillo Velasco‐Herrera, Louise van der Weyden, Jeremie Nsengimana, Anneliese O. Speak, Marcela K. Sjöberg, David Timothy Bishop, Göran Jönsson, Julia Newton‐Bishop, David J. Adams

**Affiliations:** ^1^ Experimental Cancer Genetics Wellcome Trust Sanger Institute Hinxton Cambridge UK; ^2^ Leeds Institute of Cancer and Pathology St James's University Hospital University of Leeds UK; ^3^ Departamento de Biología Celular y Molecular Facultad de Ciencias Biológicas Pontificia Universidad Católica de Chile Santiago Chile; ^4^ Division of Oncology and Pathology Department of Clinical Sciences Skåne University Hospital Lund University Sweden

**Keywords:** comparative genomics, CRISPR, melanoma, RNA‐Seq

## Abstract

Metastasis is the leading cause of death in patients with advanced melanoma, yet the somatic alterations that aid tumour cell dissemination and colonisation are poorly understood. Here, we deploy comparative genomics to identify and validate clinically relevant drivers of melanoma metastasis. To do this, we identified a set of 976 genes whose expression level was associated with a poor outcome in patients from two large melanoma cohorts. Next, we characterised the genomes and transcriptomes of mouse melanoma cell lines defined as weakly metastatic, and their highly metastatic derivatives. By comparing expression data between species, we identified lunatic fringe (*LFNG)*, among 28 genes whose expression level is predictive of poor prognosis and whose altered expression is associated with a prometastatic phenotype in mouse melanoma cells. CRISPR/Cas9‐mediated knockout of *Lfng* dramatically enhanced the capability of weakly metastatic melanoma cells to metastasise *in vivo,* a phenotype that could be rescued with the *Lfng* cDNA. Notably, genomic alterations disrupting *LFNG* are found exclusively in human metastatic melanomas sequenced as part of The Cancer Genome Atlas. Using comparative genomics, we show that *LFNG* expression plays a functional role in regulating melanoma metastasis.

**Abbreviations**
Bp: base pair
CNV: copy number variants
CRISPR: clustered regularly interspaced short palindromic repeats
DASL: cDNA‐mediated Annealing, Selection, Ligation
Dll3: Delta‐like ligand‐3
DMEM: Dulbecco's modified Eagle's medium
FDR: false discovery rate
FPKM: fragments per kilobase per million
HDC: histidine decarboxylase
HR: hazard ratio
Kb: kilobase
LFNG: lunatic fringe
MSS: melanoma‐specific survival
NDRG2: N‐myc downstream‐regulated gene 2
NICD: Notch intracellular domain
OS: overall survival
qPCR: quantitative polymerase chain reaction
RT‐PCR: reverse transcription polymerase chain reaction
SD: standard deviation
SNV: single nucleotide variation
TCGA: The Cancer Genome Atlas
TPM: Transcripts per million
UV: ultraviolet

## 1 Introduction

Melanoma is an aggressive cancer that develops from the pigment‐producing cells of the skin. In melanoma, as in other cancers, metastasis accounts for the majority of the mortality of patients with advanced disease (Chaffer and Weinberg, 2011; Damsky *et al*., 2010). This complex multistep process requires melanoma cells to invade adjacent tissues, intravasate into the lymphatics or blood vasculature, extravasate at distant sites and ultimately colonise an organ or tissue. For this to happen, melanoma cells must evade the immune system and sculpt the host microenvironment (Fidler, 2003).

Several models of metastasis have been proposed including those that describe monoclonal and polyclonal seeding. It is also clear that once a cell has left the primary tumour, it may undergo further evolution (Turajlic and Swanton, 2016). This complex pattern of tumour cell dissemination and ongoing evolution complicates the identification of the genetic events that drive the metastatic process. Importantly, transcriptome profiling of primary tumours has identified expression changes shown to be predictive of metastasis (Paik *et al*., 2004; van de Vijver *et al*., 2002), and alterations found in metastases have been shown to be present in subclones in early primary lesions (Wardwell‐Ozgo *et al*., 2013). These data support the idea that a proportion of cells within primary tumours may evolve, acquire or have intrinsic metastatic capabilities. Identifying those patients with tumours at high risk of metastasising could help identify individuals who may benefit from adjuvant therapies or more regular screening (Eggermont, 2016).

In this study, we set out to identify clinically relevant genes that confer enhanced metastatic capabilities upon melanoma cells. To do this, we used comparative functional genomics applied to gene expression predictors of patient survival, combined with expression data from murine cell line models that have different capabilities to colonise the lung, a major site of human melanoma metastasis. In this way, we identified a set of 28 genes associated with patient outcome that were also differentially expressed when weakly and highly metastatic mouse melanoma lines were compared. We focused on lunatic fringe (*LFNG*) that encodes for a glycosylating enzyme (O‐fucosylpeptide 3‐beta‐N‐acetylglucosaminyltransferase) that regulates NOTCH signalling (Moloney *et al*., 2000), and show an important role for this gene in controlling melanoma metastasis.

## 2 Materials and methods

### 2.1 Survival analysis

Gene expression data generated using whole‐genome cDNA‐mediated annealing, selection, ligation and extension (DASL) arrays (Illumina Inc., San Diego, CA, USA) from 217 (Leeds) (Nsengimana *et al*., 2015) and 222 (Lund) (Jonsson *et al*., 2010) primary melanomas (209 cutaneous, 13 mucosal) were obtained. The Leeds data set (Leeds melanoma cohort, *N* = 204, and chemotherapy study, *N* = 13) was profiled on the human HT12.4 array, while the Lund cohort was profiled on the earlier HT8.3 version. Quality control and normalisation of these data sets has been published elsewhere (Jonsson *et al*., 2010; Nsengimana *et al*., 2015). Briefly, the HT8.3 version had a lower performance with only 7752 genes passing QC filters. The overlap between this set and the Leeds data set using HT12.4 was 7584 genes. Survival benefit of each gene (log_2_ scale) was assessed in a Cox proportional hazards model using STATA v14.2 (STATACorp, Texas, USA) for melanoma‐specific survival (MSS) in the Leeds data and overall survival (OS) using the data from Lund. Analysis of the Leeds data set was adjusted for patient age and sex. *P*‐values were corrected for multiple testing (Benjamini–Hochberg false discovery rate, FDR). Kaplan–Meier curves were plotted comparing high to low gene expression relative to the median. Functional gene annotation and enrichment analyses of the genes that showed the same direction of association in both patient cohorts were performed using DAVID (Huang *et al*., 2009).

### 2.2 Cell lines

B16‐F0 and B16‐F10 cell lines were purchased from the American Type Culture Collection (ATCC), and the B16‐BL6, K1735‐P and K1735‐M2 lines were obtained from the University of Texas M.D. Anderson Cancer Centre. All cell lines were screened for the presence of mycoplasma and other mouse pathogens (Charles River Laboratories, Wilmington, MA, USA). Cells were cultured at 37°C in 5% CO_2_ in high glucose Dulbecco's modified Eagle's medium (DMEM) supplemented with 10% fetal bovine serum, 29.2 mg·mL^−1^ L‐glutamine, 10 000 units·mL^−1^ penicillin and 10 000 μg·mL^−1^ streptomycin.

### 2.3 Nucleic acid extraction and sequencing

For whole‐genome sequencing, DNA was extracted from cell pellets using the QIAGEN Puregene Core Kit A. Paired‐end 75‐bp libraries were prepared and sequenced using the Illumina HiSeq platform. Data have been deposited in the European Nucleotide Archive (ERP001691). RNA was extracted from cell pellets using the QIAGEN RNeasy Mini Kit. Five different vials were cultured and extracted per cell line, to obtain five independent biological replicates for each line. 1 μg of total RNA per sample was submitted for sequencing. Unstranded 75‐bp paired‐end barcoded libraries were prepared with the standard Illumina library preparation kit. RNA libraries were sequenced on the Illumina Hiseq platform and the data deposited in public databases (European Nucleotide Archive (ERP001690) and ArrayExpress (E‐ERAD‐94)).

### 2.4 Whole‐genome data processing and somatic variant calling

Raw reads were mapped to the mouse reference genome (GRCm38p1) using bwa‐mem (Li, 2013) v0.7.5 and PCR duplicates marked using Picard tools MarkDuplicates v1.72 (http://broadinstitute.github.io/picard). Single nucleotide variants (SNVs) and short indels were called using Samtools mpileup (Li *et al*., 2009) v0.1.19‐58‐g3d123 cd, and the resulting variants were filtered using VCFTools (Danecek *et al*., 2011). Variants with variant quality QUAL < 20, or number of reads supporting the variant less than 5 (DP < 5) or SNPGAP < 10, were discarded. Due to the absence of a matched germline/normal sample from the exact mouse from which the cell lines were generated, we removed all variants reported by the mouse genomes project (Keane *et al*., 2011) for the genetic backgrounds of each cell line group. Similarly, variants located within ± 50 bp of structural variants reported by the mouse genomes project (Keane *et al*., 2011) were also discarded. Finally, functional consequences were predicted using ENSEMBL's variant effect predictor (v74) (McLaren *et al*., 2016).

### 2.5 Orthogonal validation of single nucleotide variants using Sequenom

A total of 262 SNVs (116 for the K1735 lines and 146 for the B16 lines) were selected for orthogonal validation using the Sequenom platform. These variants were randomly chosen using GATK's ValidationSiteSelector (v2.8‐1‐g932cd3a) from the set of variants that were identified to be present in all the cell lines from each group. All assays using the Sequenom platform were performed with three biological replicates of each line.

### 2.6 Somatic signature identification and comparison

Somatic mutational signatures were identified for each mouse cell line group using the filtered somatic single nucleotide variants (above). Signatures were identified using the non‐negative matrix factorisation method from the SomaticSignatures R package (Gehring *et al*., 2015) (v 2.6.1). To compare these signatures to those reported in COSMIC (http://cancer.sanger.ac.uk/cancergenome/assets/signatures_probabilities.txt), we calculated cosine similarities as previously reported (Alexandrov *et al*., 2015).

### 2.7 Copy number calling

Copy number alterations were identified using Control‐FREEC (Boeva *et al*., 2011) v6.7 with 50‐Kb windows. Due to the lack of a matched normal for each cell line, CNVs were called relative to parental cell lines (B16‐F0 and K1735‐P); somatic CNVs for the B16‐BL6 line were called using the BAM file for B16‐F10.

### 2.8 Identification of differentially expressed genes

Raw paired‐end reads were aligned to the mouse reference genome (GRCm38p1) using the splice‐aware aligner Tophat2 (Kim *et al*., 2013) guided by ENSEMBL mouse annotation (v73). Subsequently, the number of uniquely mapped read pairs that were aligned to each gene within the annotation with a mapping quality > 10 were counted using htseq‐count (Anders *et al*., 2015). Raw counts were normalised by calculating the fragments per kilobase per million (FPKM) values for each gene for each replicate. As a ‘fit for use’ quality control, blind pairwise comparisons across all the RNA‐Seq samples were performed by calculating the Pearson's correlation coefficient based on the FPKM values of all protein‐coding genes of the 25 sequenced samples. This information was used to group the samples using unsupervised hierarchical clustering using the package gplots in R (Gregory *et al*., 2013). To identify differentially expressed genes, all of the four possible paired comparisons between cell lines and their more metastatic derivatives were made (B16‐F10 vs B16‐F0, B16‐BL6 vs B16‐F10, B16‐BL6 vs B16‐F0 and K1735‐M2 vs K1735‐P) using DESeq2 (Love *et al*., 2014). Once dispersion estimates and normalised counts were calculated, genes with mean normalised counts < 10 were filtered out and *P*‐values were re‐adjusted using the Benjamini–Hochberg correction for multiple testing. All genes with *P* < 0.01 and a log_2_(foldchange) ≤−2 or ≥ 2 were considered as differentially expressed.

### 2.9 Mouse–human orthologue identification

To identify the human orthologues of mouse genes, the Compara module from the ENSEMBL Perl API was used (Herrero *et al*., 2016). In cases where a mouse gene had multiple orthologues in humans, the gene with the highest percentage of identity when comparing the human and mouse proteins was selected. Genes that had an ortholog classification of ‘many2many’ were not considered for further analysis.

### 2.10 Randomisation test to identify the expected number by chance of differentially expressed mouse genes overlapping and concordant with the list of genes associated with poor survival in melanoma patients

Two independent randomisation tests were performed using two different sample sizes 1290 or 388. A total of 1000 samples with randomly selected mouse genes (out of the 15 412 genes with normalised fragment counts > = 10 expressed by B16‐F0 or B16‐BL6) of each sample size were generated. Genes were selected without replacement. For each randomly selected gene, a random direction of expression was assigned with the same probability as the one observed in the mouse data: underexpressed (0.4621429) or overexpressed (0.5378571). Then, each random sample was compared to the list of human genes associated with poor outcome with an FDR < 0.1 in our combined patient survival analysis, to identify the number of overlapping and concordant genes. Finally using the distribution of the number of overlapping and concordant genes across the 1000 samples, we calculated the probability of obtaining a number of overlapping genes or more as the one observed in the mouse cell line/human data comparison.

### 2.11 Cas9 gRNA selection

To select suitable gRNAs, we identified sequences in the exons of candidate genes in the ENSEMBL v71 annotation of the GRCm38 mouse reference genome (′5‐NNNNNNNNNNNNNNNNNNNNNGG‐3′). For each sequence, possible off‐targets were identified using Cas‐Offinder (Bae *et al*., 2014). We then used biomaRt (Durinck *et al*., 2009) to identify all possible off‐targets with up to three mismatches whose expected cutting site overlapped an exon. Targeting sequences with zero exonic off‐targets with up to three mismatches were selected. See Table S7.

### 2.12 *Lfng* disruption using a single gRNA (*g2d1* clone generation)

Oligos with the *Lfng* gRNA sequence (Sigma‐Aldrich Corp, St. Louis, MO, USA) were cloned into the vector PX459 (Addgene #48139) following the Zhang laboratory protocol (Ran *et al*., 2013). Plasmids were validated by Sanger sequencing using a U6 oligo (Table S7). To obtain stable transfectants, the region containing the U6 promoter, gRNA, gRNA scaffold and the CBh‐hSpCsn1‐PURO‐PolyA was excised and cloned into the PiggyBac plasmid PB713B‐1 to make PX459_Lfng_g2_gRNA‐PB713B‐1 (Fig. S12B). B16‐F0 cells (6 × 10^5^) were cotransfected with 0.5 μg pCMV‐PiggyBac PBase (System Biosciences) and 5 μg of either PX459_Lfng_g2_gRNA‐PB713B‐1 plasmid (to generate Lfng‐targeted cells) or empty PB713B‐1 plasmid (to generate ‘control’ cells) using Fugene HD (Promega Corporation, Madison, WI, USA). Twenty‐four hours later, 5 μg puromycin was added to the medium and after 7 days individual colonies were isolated. Sequences amplified from the *Lfng* locus were analysed with TIDE (Brinkman *et al*., 2014) to identify clones with disruptive mutations. Clone ‘*g2d1*’, carrying a homozygous 1‐bp insertion in *Lfng*, and clone ‘*ca4*’ (from the control plate) were selected for further analysis.

### 2.13 *Lfng* disruption using two gRNAs (*L1* clone generation)

Oligos with the *Lfng* targeting sequences (Sigma‐Aldrich Corp) were cloned into the PiggyBac gRNA expressing vector, Piggy_gRNAScaffold_BLASTO (S12B), following the Zhang's laboratory protocol (Ran *et al*., 2013). Plasmids carrying gRNA sequences were validated by Sanger sequencing using a U6 oligo (Table S7). To target *Lfng* using two different gRNA sequences (gRNAs Lfng_g2 and Lfng_g3; Table S7), we first generated a Cas9 stably expressing B16‐F0 cell line by cotransfecting 6 × 10^5^ B16‐F0 cells with 5 μg of pPB‐LR5.1‐EF1a‐puro2ACas9 (Koike‐Yusa *et al*., 2014) and 0.5 μg pCMV‐piggyBac. From this experiment, we cloned a single cell line and cotransfected 6 × 10^5^ cells with 2.5 μg of Piggy_gRNAscaffold_Lfng_g2 and 2.5 μg of Piggy_gRNAscaffold_Lfng_g3 (Fig. S12B), or LMDJ‐Piggy_gRNAscaffold to generate a control cell line. Twenty‐four hours later, 10 μg blasticidin was added to the medium, and after 7 days, individual colonies were isolated and assessed for targeting of *Lfng* by PCR. Clone ‘*L1*’, carrying a 4.8‐kb deletion encompassing exons 1–4 of *Lfng*, and clones ‘*C1*’ and ‘*C2*’ (from the control plate) were selected for further analysis.

### 2.14 *Lfng* cDNA rescue experiments

To confirm that the metastasis phenotypes we observed were due to the disruption of *Lfng,* we used plasmid rescue in the *L1* cell line using the vector PB533A‐2 carrying a flag‐tagged full‐length *Lfng* cDNA (synthesised by GeneArt) to generate the cell line *L1‐Lfng*. *L1‐PB* cells carrying the empty vector were used as a control.

### 2.15 Assessment of *Lfng* expression in cell lines by quantitative RT‐PCR

For the comparison of *Lfng* expression levels between cell lines, RNA was extracted from 1 × 10^6^ cells using the RNAeasy mini Kit (QIAgen, Manchester, UK) and cDNA was prepared using the SuperScript VILO Master Mix (Thermo) according to the manufacturers’ instructions. RT‐qPCR was performed using the TaqMan Fast Advanced Master Mix. *Lfng* (Mm01201988_m1) and *B2m* (Mm00437762_m1) assays were used for these studies. Reactions were performed in quadruplicate using the StepOnePlus system (Thermo Fischer Scientific, Waltham, MA, USA), and analysis was performed using the ΔΔCt method (Schmittgen and Livak, 2008).

### 2.16 Western blotting

Western blotting was performed using standard approaches. Anti‐vinculin (clone V284) and anti‐Flag (clone M2) antibodies were used (Sigma‐Aldrich Corp).

### 2.17 *In vivo* experimental metastasis assays

The experimental metastasis assay was performed as described previously (van der Weyden *et al*., 2017). For testing of the K1735‐P and K1735‐M2 cell lines, 1 × 10^5^ cells were tail‐vein‐dosed into six‐ to eight‐week‐old wild‐type C3H/HeJ mice. After 10 days, mice were humanely sacrificed and their lungs were collected into 10% neutral buffered formalin and then processed for histopathological analysis. For testing of the B16‐F0, B16‐F10, and B16‐BL6 cell lines, 0.75 × 10^5^ cells were tail‐vein‐dosed into six‐ to eight‐week‐old wild‐type C57BL6/NTac mice and their pulmonary metastatic burden was determined 7 days later by macroscopic counting. For testing the *Lfng*‐targeted *g2d1* cells (and respective *ca4* control cells), 4 × 10^5^ cells were tail‐vein‐dosed, and for testing the *Lfng*‐targeted *L1* cells (and respective C1/C2 control cells), 5 × 10^5^ cells were tail‐vein‐dosed; both into six‐ to eight‐week‐old wild‐type C57BL/6NTac mice. cDNA rescue experiments, using the cell line *L1‐Lfng* and the control *L1‐PB*, were performed using 4 × 10^5^ cells. The pulmonary metastatic burden was determined 10 days postdosing by macroscopic counting. In all cases, sex‐matched mice were used. The care and use of all mice in this study was in accordance with the Animals (Scientific Procedures) Act 1986 Amendment Regulations 2012, and all procedures were performed under a UK Home Office Project licence (PPL 80/2562). All mice were housed in individually ventilated cages (Techniplast GM500) receiving 60 air changes per hour, in a specific pathogen‐free environment with *ad libitum* access to autoclaved water and food (Mouse Breeders Diet, Laboratory Diets, 5021‐3). Cages were filled with aspen bedding substrate, with a nestlet and fun tunnel for environmental enrichment. There was a 12‐h light/dark cycle with no twilight period with a temperature of 21 °C ± 2 °C and a humidity of 55% ± 10%. Throughout the experiment, the welfare of the mice was monitored with daily visual checks.

### 2.18 Whole exome sequencing of the *L1* cell line

DNA from *L1* cells was exome‐sequenced using Agilent mouse whole exome baits. A 75‐bp paired‐end library was prepared and sequenced on the Illumina HiSeq2500 platform. Data were analysed as above and are available in the European Nucleotide Archive (ERP015062).

## 3 Results

### 3.1 mRNA expression predictors of prognosis in primary melanoma

**Figure 1:**
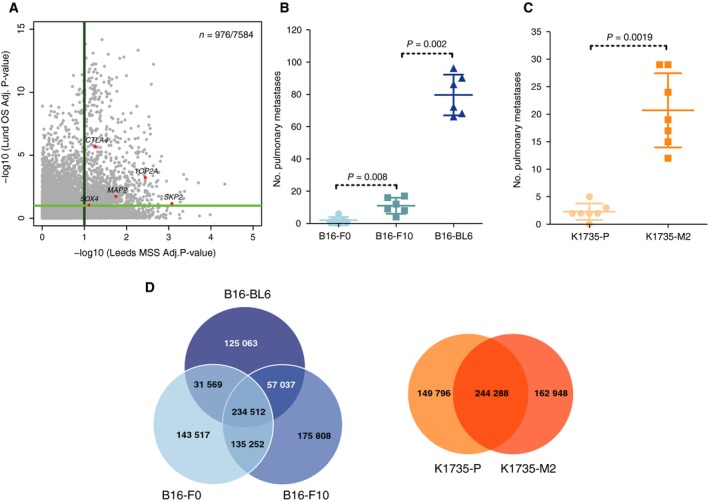
Patient sample and mouse cell line characteristics**.** (A) Scatter plot showing the −log_10_‐corrected *P*‐values for the 7584 genes analysed in both cohorts in association with melanoma‐specific survival in the Leeds cohort (*x*‐axis) and overall survival in the Lund cohort (*y*‐axis). (B‐C) Experimental metastasis assay using (B) B16 cell lines and (C) K1735 cell lines in wild‐type female mice (symbols representing individual mice with horizontal bar at the mean ± SD and statistics performed using a Mann–Whitney test; data shown are representative of two independent experiments). (D) Venn diagrams showing the number of variants shared between the mouse melanoma cell lines.

Both tumour depth (Breslow thickness) and ulceration are established predictors of melanoma metastasis (Nsengimana *et al*., 2015), but the underlying mechanisms that drive metastasis are unknown. We first set out to identify genes whose expression levels were associated with poor outcome. To do this, we analysed the expression profiles of primary melanomas from two previously published studies from Leeds (the Leeds Melanoma Cohort and chemotherapy studies, *n* = 217) and from the Lund Melanoma Research Group (*n* = 222) (Jonsson *et al*., 2010; Nsengimana *et al*., 2015). Demographic information for these cohorts is provided in Table S1. Tumours from both cohorts have been analysed using Illumina DASL arrays such that the expression of 7584 genes may be assessed. For the Leeds cohort, melanoma‐specific survival (MSS) data were available, whereas overall survival (OS) was recorded for the Lund cohort. Survival analyses stratifying by gene expression were performed using the Cox proportional hazards model. In this way, we identified 976 genes whose expression levels were significantly associated with patient outcome in both cohorts (FDR < 0.1; Fig. 1A, Table S2). Of these genes, 78.17% (763/976) showed the same direction of association in both cohorts. These genes included *SKP2* (Chen *et al*., 2011), *TOP2A* (Song *et al*., 2013), *SOX4* (Jafarnejad *et al*., 2010), *MAP2* (Soltani *et al*., 2005) and *CTLA4* (Hannani *et al*., 2015), all of which have been associated with patient outcome in melanoma. Gene enrichment analysis found that biological processes including epidermis development, keratinocyte differentiation and immune response were overrepresented (FDR < 0.01; Table S3) – biological processes previously reported to be important in the development of melanoma metastasis (Bald *et al*., 2014; Golan *et al*., 2015).

### 3.2 Genomic characterisation of murine melanoma cell lines with contrasting metastatic capabilities

**Figure 2:**
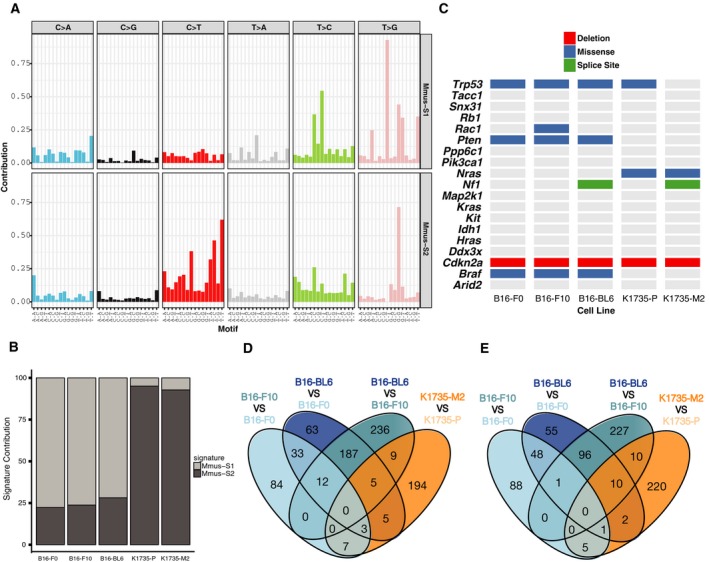
Characterisation of mouse melanoma cell line series. (A) Somatic mutational signatures operative in the genomes of mouse melanoma cell lines. (B) Signature contribution in the mouse melanoma cell line genomes for each process identified. (C) Matrix showing the mutations in known melanoma driver genes found in mouse melanoma cell lines. Venn diagrams showing the number of genes identified as differentially (D) overexpressed or (E) underexpressed across the multiple paired comparisons between a cell line with higher metastatic potential and its parental line.

To facilitate comparative analyses, we selected five mouse melanoma cell lines with different metastatic capabilities: B16‐F0, B16‐F10 and B16‐BL6 derived from C57BL/6 mice and K1735‐P and K1735‐M2, derived from the C3H strain (Fidler, 1970, 1973; Kripke, 1979; Kripke *et al*., 1978). Prior to genomic analysis, we validated the metastatic capabilities of these lines *in vivo* using an experimental metastasis assay (Fig. 1B–C). Consistent with previous reports (Poste *et al*., 1980; Talmadge and Fidler, 1982), B16‐BL6 cells were highly metastatic when compared to B16‐F10 or B16‐F0 cells and K1735‐M2 cells were highly metastatic when compared to K1735‐P cells. Spectral karyotyping of these cell lines showed high levels of polyploidy and multiple chromosomal aberrations (Figs. S1‐S2 and Table S4). We sequenced each of these lines to 30‐56x whole‐genome coverage, using the Illumina HiSeq platform. To identify somatic mutations (SNVs), we mapped these data to the reference C57BL/6J genome (GRCm38) and filtered the calls using variants described by the Mouse Genomes Project (Keane *et al*., 2011) and for quality (as detailed in Fig. S3). The number of variants shared among the lines within the B16 and K1735 groups is shown in Fig. 1D. The B16 lines showed higher numbers of somatic SNVs and short indels (~ < 50 bp) than the K1735 lines, with an average of 267 566 and 243 913 SNV, respectively (Fig. S4). A copy number analysis was also performed (Fig. S5 and Table S5). To assess our variant calling, we randomly selected 262 variants for validation by Sequenom genotyping (146 identified from the B16 cell lines and 116 from the K1735 lines), obtaining an overall validation rate of 90.86% for the B16 lines and 76.72% for the K1735 lines (Fig. S6). In B16 cell lines, the predominant mutation type was T > G (Fig. 2A) and the predominant mutational signature was Mmus‐S1 (Fig. 2B), which shows highest similarity to human mutational signature, signature 17 (Alexandrov *et al*., 2013) (cosine similarity 0.872) – a signature whose aetiology is currently unknown but has been observed in melanoma tumours (http://cancer.sanger.ac.uk/cosmic/signatures). In K1735 cell lines, the predominant mutational signature was Mmus‐S2 (Fig. 2B), with similarity to the UV light signature reported by COSMIC (signature 7; cosine similarity 0.597), which is in keeping with the genesis of these lines following the combined administration of UV light and croton oil (Kripke, 1979; Kripke *et al*., 1978; Talmadge and Fidler, 1982). Further characterisation revealed that both K1735 cell lines carried a homozygous activating mutation in *Nras* (p.G13D) and deletion of the first exon of *Cdkn2a* (*p19* gene*)*, as previously reported (Melnikova *et al*., 2004) (Fig. S7), as well as an unreported *Trp53* mutation (p.T74P) in K1735‐P cells (Fig. 2C). B16 cell lines carried a deletion of the entire *Cdkn2a* locus*,* as previously reported (Melnikova *et al*., 2004) (Fig. S7), as well as an unreported heterozygous missense mutation in *Braf* (p.C263R; predicted to be deleterious by SIFT), a missense *Trp53* mutation (p. N125D) and a mutation in *Pten (*p.T131P; Fig. 2C). Finally, we could observe that mutations in *Rac1* and *Nf1* were only present in the more invasive derivative lines. For instance, the *Rac1* missense mutation (p.A59S) was present only in B16‐F10 cells. Similarly, the splice site variant (Chr11.79408779T>G) within *Nf1*, was observed in B16‐BL6 and K1735‐M2.

### 3.3 Transcriptomic characterisation of murine melanoma cell lines

We generated RNA‐seq data from five biological replicates for each of the five melanoma cell lines. We mapped the reads against the GRCm38 mouse reference genome, counted the number of read pairs and verified the correlation among biological replicates (*r* > 0.95; Fig. S8). In an effort to identify changes in RNA levels that associate with higher metastatic capabilities, we identified all genes that were differentially expressed between the parental lines (B16‐F0, K1735‐P) and their more metastatic derivatives (B16‐F10, B16‐BL6 and K1735‐M2). To do this, we performed all possible paired comparisons within each group using DESeq2 (Love *et al*., 2014). Genes were classified as differentially expressed if their *P*‐value, after multiple testing correction, was *P*‐adj < 0.01, with an expression change of fourfold or more. In this way, we identified a total of 1430 genes that were differentially expressed (Table S6). qPCR was performed on selected genes for validation (Fig. S9). Notably, no genes were consistently differentially expressed across the comparisons of B16 and K1735 parental lines to their more metastatic derivatives (Fig. 2D–E), suggesting that different mechanisms confer metastatic potential in these cell lines series.

### 3.4 Identification of conserved putative regulators of metastatic colonisation in melanoma

**Figure 3:**
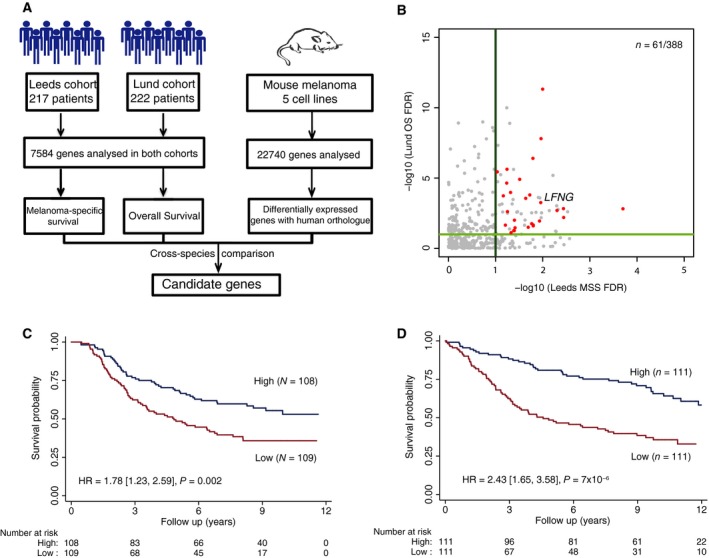
Cross‐species metastasis colonisation gene candidate identification. (A) Diagram showing the cross‐species approach used to identify gene candidates. (B) Scatter plot showing the corrected *P*‐values (−log_10_) obtained from the survival analysis for the 388 genes that can be analysed in both human patient cohorts and whose orthologue in mouse were identified as differentially expressed in metastatic cell lines. In red, genes with concordance between the expression changes in the mouse cell lines comparisons and the gene levels associated with poor outcome in both patient cohorts with an FDR < 0.1 are shown. (C) Kaplan–Meier curve showing the melanoma‐specific patient survival in the Leeds cohort when stratified by *LFNG* expression. (D) Kaplan–Meier curve showing the overall patient survival in the Lund cohort when stratified by *LFNG* expression. The plots show the results when the data are stratified by median expression into high and low *LFNG* expression groups. The hazard ratio shown is for the low expression group vs the high expression group.

To identify putative regulators of metastatic colonisation in melanoma, we next took a comparative genomics approach (Fig. 3A). We identified the human orthologues for all 1430 differentially expressed genes identified from the mouse melanoma cell line comparisons. For each mouse gene, we selected the orthologue with the highest protein sequence identity between mouse and humans. All paralogous genes were discarded. These criteria retained 1290 of the 1430 differentially expressed genes. We intersected these genes with the 7584 genes analysed in both human cohorts, which left 338 genes; 61 of which were significantly predictive of survival in both human cohorts (FDR < 0.1). Of these 61 genes, 28 genes showed the same direction of expression change (up‐ or downregulation) in relation to poor patient outcome and cell line metastasis phenotype (Table 1, Fig. 3B). A summary of the gene numbers obtained through each stage of our analysis is presented in Fig. S10. To assess the statistical significance of this result, we performed two independent randomisation tests revealing that the probability of obtaining 28 concordant genes by chance when intersecting a gene set with the human survival data was *P* (*x* ≥ 28) = 0.024 (when *n* = 1290) and *P* (*x* ≥ 28) = 0 (when *n* = 388) (Fig. S11).

**Table 1:** Candidate genes identified in this study. Genes identified to be associated with metastasis and poor patient prognosis. This table shows gene expression on a continuous scale. The hazard ratios (HR) shown are per each additional unit of log_2_ gene expression

Gene	Leeds cohort Melanoma‐Specific Survival				Lund cohort overall survival			
	Hazard Ratio	95% C.I.	*P*‐val	FDR	Hazard Ratio	95% C.I.	*P*‐val	FDR
*CD82*	0.58415	0.48411, 0.70485	2.03E‐08	0.00020	0.52520	0.37432, 0.73692	0.00019	0.00153
*LFNG*	0.60248	0.45858, 0.79153	0.00027	0.01102	0.52328	0.38240, 0.71605	5.18E‐05	0.00055
*PTK2B*	0.64250	0.52325, 0.78893	2.41E‐05	0.00364	0.47279	0.31916, 0.70039	0.00019	0.00148
*CCL5*	0.67761	0.56575, 0.81157	2.35E‐05	0.00360	0.57686	0.41392, 0.80393	0.00116	0.00629
*LSP1*	0.68714	0.57354, 0.82325	4.71E‐05	0.00497	0.48760	0.33130, 0.71764	0.00027	0.00201
*DDX60*	0.71537	0.59150, 0.86520	0.00056	0.01594	0.53727	0.34560, 0.83524	0.00578	0.02156
*PARP14*	0.71561	0.58722, 0.87208	0.00091	0.02035	0.57359	0.37679, 0.87319	0.00953	0.03145
*TUBA4A*	0.71722	0.60117, 0.85567	0.00022	0.01002	0.31663	0.23623, 0.42440	1.42E‐14	4.79E‐12
*GPX3*	0.71882	0.60071, 0.86016	0.00031	0.01170	0.26009	0.10878, 0.62186	0.00246	0.01130
*NDUFA4L2*	0.72297	0.60147, 0.86901	0.00055	0.01588	0.67309	0.50482, 0.89746	0.00700	0.02488
*GABRE*	0.72349	0.59532, 0.87925	0.00114	0.02295	0.42004	0.28150, 0.62674	2.16E‐05	0.00028
*BTBD6*	0.72365	0.58391, 0.89682	0.00313	0.04005	0.47218	0.25171, 0.88574	0.01938	0.05445
*ITM2A*	0.72406	0.60252, 0.87011	0.00057	0.01613	0.35698	0.25098, 0.50776	1.00E‐08	3.96E‐07
*HDC*	0.73176	0.61012, 0.87764	0.00076	0.01881	0.63449	0.51806, 0.77708	1.09E‐05	0.00016
*ELF4*	0.73257	0.61273, 0.87584	0.00064	0.01716	0.56910	0.38619, 0.83865	0.00438	0.01738
*RIPK3*	0.73743	0.62606, 0.86861	0.00027	0.01085	0.40256	0.30353, 0.53388	2.67E‐10	1.57E‐08
*CCBE1*	0.73817	0.59969, 0.90862	0.00418	0.04704	0.35930	0.14251, 0.90584	0.03004	0.07619
*PON3*	0.74237	0.61458, 0.89673	0.00200	0.03083	0.64187	0.53994, 0.76304	5.02E‐07	1.23E‐05
*BOC*	0.74269	0.61053, 0.90345	0.00292	0.03872	0.67546	0.50016, 0.91219	0.01048	0.03398
*RUNX1T1*	0.74951	0.61916, 0.90730	0.00310	0.03987	0.57779	0.40754, 0.81917	0.00207	0.00983
*FILIP1L*	0.76881	0.64177, 0.92100	0.00433	0.04808	0.49836	0.36807, 0.67478	6.67E‐06	0.00011
*NDRG2*	0.77243	0.64328, 0.92751	0.00567	0.05603	0.56030	0.40811, 0.76923	0.00034	0.00241
*TSPAN33*	0.77331	0.65148, 0.91792	0.00329	0.04124	0.46715	0.24663, 0.88486	0.01953	0.05474
*FAM110C*	0.77589	0.64552, 0.93259	0.00686	0.06188	0.62169	0.44282, 0.87281	0.00603	0.02221
*JUP*	0.77882	0.65184, 0.93053	0.00591	0.05722	0.61515	0.51528, 0.73437	7.62E‐08	2.36E‐06
*RUNX3*	0.78039	0.64937, 0.93784	0.00818	0.06857	0.34593	0.21468, 0.55742	1.29E‐05	0.00018
*EGLN3*	0.78592	0.64904, 0.95166	0.01361	0.09212	0.42935	0.31385, 0.58735	1.23E‐07	3.61E‐06
*MID1*	1.37339	1.09496, 1.72262	0.00606	0.05803	3.45380	2.09940, 5.68197	1.06E‐06	2.28E‐05

Of the above‐mentioned 28 genes, only one was upregulated in poor outcome patients, specifically *MID1*. Notably, 5 of 28 genes we identified have previously been reported to affect melanoma metastasis: *CD82, NDRG2, RUNX3, CCL5 and HDC*. For example, reports suggest that CD82 expression in melanoma cells inhibits tumour cell extravasation and lung metastasis formation *in vivo* (Khanna *et al*., 2014); upregulation of CD82 predicted better outcome in our analysis of two independent cohorts (Table 1). In addition to this, the tumour suppressor N‐myc downstream‐regulated gene 2 (*NDRG2)* is known to restrict melanomagenesis by regulating *Mitf* expression (Kim *et al*., 2008). Similarly, the tumour suppressor *RUNX3* has been shown to be downregulated in metastatic melanoma lines when compared to primary melanoma or healthy skin (Kitago *et al*., 2009), while expression of the chemokine and leucocyte chemoattractant CCL5 in B16 cells strongly suppresses lung metastasis (Aravindaram *et al*., 2009). Finally, the histidine decarboxylase (*HDC*) gene encodes a protein whose function is to convert L‐histidine to histamine. Histamine has been shown to play an important role in immune cell function (Hansson *et al*., 1999), and a histamine/IL‐2 combination has been used to increase T‐cell responses in stage IV melanoma patients (Asemissen *et al*., 2005). In addition to the five genes mentioned above, an additional nine genes *(CCBE1, GPX3, PTK2B, LSP1, DDX60, PARP14, GABRE, JUP* and *MID1)* have been associated with metastasis in other types of epithelial or solid cancers (Aktary and Pasdar, 2013; Bachmann *et al*., 2014; Hao *et al*., 2010; Jeltsch *et al*., 2014; Koral *et al*., 2015; Yue *et al*., 2015; Zhang *et al*., 2014b).

Next, we ranked the 28 identified genes based on their hazard ratios and overall patient survival, as well as their *P*‐values after multiple test correction (Table 1). Based on these criteria, we selected the gene *LFNG* for further analysis.

Lunatic fringe (LFNG) is a glycosylating enzyme that post‐translationally modifies Notch receptor proteins (LeBon *et al*., 2014). LFNG‐mediated glycosylation of Notch receptors alters the binding affinity of Notch proteins with their ligands and activation of Notch receptors by delta‐like ligands (Kakuda and Haltiwanger, 2017). In our analyses, low RNA levels of *LFNG* were shown to be associated with poor outcome in both patient cohorts (Fig. 3C–D). Moreover, *LFNG* is prognostic for melanoma‐specific survival independent of Breslow thickness. Furthermore, B16‐BL6 cells had decreased *Lfng* expression when compared to its B16‐F0 parental line (log_2_(foldchange) = −2.185773, *P* = 1.069061 × 10^−6^, negative binomial Wald test with Benjamini–Hochberg correction), which was confirmed by qPCR (Fig. S12A).

### 3.5 *Lfng* disruption enhances the lung colonisation capabilities of CRISPR/Cas9‐targeted melanoma cells

**Figure 4:**
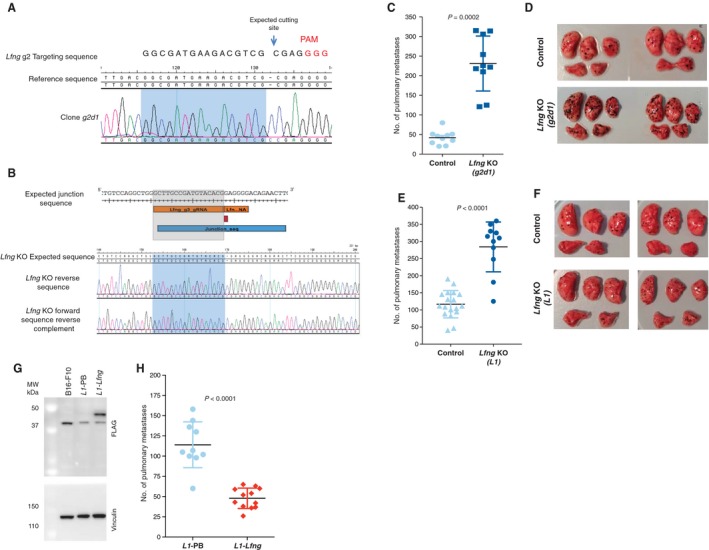
*In vivo* validation of the role of *Lfng* in metastasis. To validate the role of *Lfng* in metastasis, two independent *Lfng* targeting experiments were performed in B16‐F0 cells: one using a single gRNA to introduce a single base pair insertion (A, C and D) and another using two gRNAs to induce a 4.8‐kb deletion (B, E and F). (A) Sanger sequence trace of the targeted region in clone *g2d1* carrying a homozygous 1‐bp insertion. (B) Image showing from top to bottom, the expected junction sequence after the deletion caused by the targeting of Lfng using two gRNAs, the expected reference sequence and the Sanger sequence traces observed and assembled with SeqMan Pro (Lasergene) against the expected reference sequence. The expected junction sequence separated by a single base insertion can be observed**.** Experimental metastasis assays using control and *Lfng*‐deficient cell lines (tail‐vein‐injected into wild‐type female mice (symbols representing individual mice with horizontal bar at the mean ± SD and statistics performed using a Mann–Whitney test; data shown are representative of two independent experiments)). Photographs are representative images of the lungs from mice injected with control and *Lfng*‐deficient cell lines. Plasmid rescue showing that introduction of the *Lfng* cDNA reverts the metastatic phenotype of *L1* cells (*L1‐Lfng*; Lfng‐transfected cells, *L1‐PB*, vector‐only controls) (G and H). (G) A western blot with an anti‐Flag antibody shows restoration of Lfng expression (clone *L1‐Lfng*). An anti‐vinculin antibody was used as a loading control. These results are representative of three independent experiments. (H) Experimental metastasis assays using control *L1‐PB* cells and Lfng‐transfected cells. Please note experiments in e and h were performed with 5 × 10^5^ and 4 × 10^5^ cells, respectively, hence the different metastasis counts.

To test the effect of *Lfng* disruption on the metastatic capabilities of melanoma cells, we used CRISPR/Cas9 to target *Lfng* in order to determine whether this may confer enhanced metastatic capabilities upon the weakly metastatic B16‐F0 cell line (Fig. S12B). We generated two independently targeted *Lfng* null B16‐F0 clones, termed *g2d1* and *L1* (Fig. 4A–B). *G2d1* cells carried a single base insertion resulting in a frameshift loss‐of‐function mutation (Fig. S13) and *L1* cells carried a 4.8‐kb deletion encompassing exons 1–4 which we verified by exome sequencing (Fig. S14). Additionally, exome sequencing identified that *L1* cells carried 413 variants (334 SNVs and 79 Indels) not present in B16‐F0 cells. Of these variants, 120 were missense, two were nonsense (altering genes *Aqp3 and Vmn2r115*) and three were frameshift mutations (affecting *Cyp7b1*,* Olfr657*, and *Vmn2r115*).

In an experimental metastasis assay, both *g2d1* cells (Fig. 4C–D) and *L1* cells (Fig. 4E–F) showed significantly increased numbers of pulmonary metastases when compared to control cells (transfected with an empty guide vector). These results directly demonstrate that *Lfng* loss enhances the metastatic capabilities of B16‐F0 cells. It was notable that compared to clone *g2d1*, clone *L1* reproducibly produced numerous smaller lung foci. To further confirm the role of *Lfng* in metastasis, we used a full‐length *Lfng* cDNA to rescue the metastatic phenotype of *L1* cells (Fig 4G–H).

### 3.6 Analysis of somatic *LFNG* mutations in human melanomas

Using two large patient cohorts (Jonsson *et al*., 2010; Nsengimana *et al*., 2015), we showed that reduced expression of *LFNG* is associated with poor patient outcome. We next evaluated the prevalence of inactivating somatic *LFNG* mutations in The Cancer Genome Atlas (TCGA) cutaneous melanoma collection (Cancer Genome Atlas Network, 2015) comparing primary vs metastatic melanoma. In this way, we identified five samples (5**/**481) with nonsilent mutations (four missense and one nonsense), all of which were in metastases. Due to short follow‐up times reported for melanoma primary tumours by TCGA, assessment of the association between *LFNG* expression and survival is not possible.

### 3.7 Analysis of NOTCH pathway gene expression in B16‐derived melanoma lines

**Figure 5:**
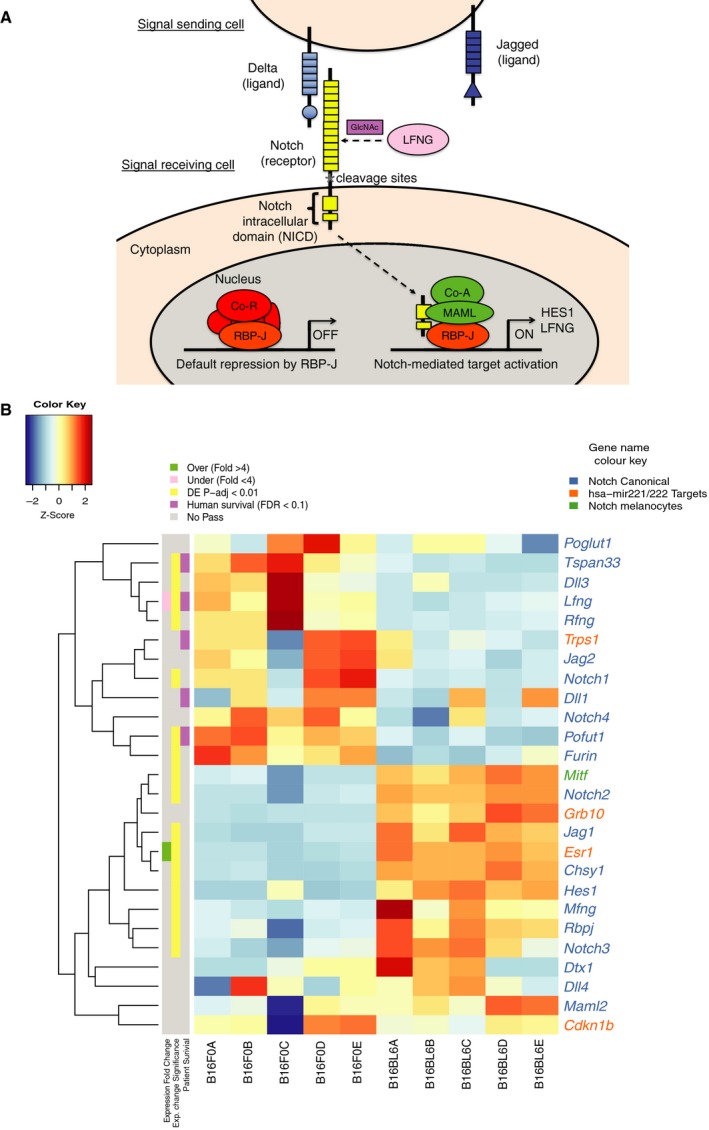
Notch pathway expression. (A) Diagram showing the canonical Notch pathway (*LFNG‐*mediated glycosylation occurs in the Golgi but is shown to depict the effect it has in mediating NOTCH–delta‐like ligand interactions). (B) Heatmap showing the *z*‐scores calculated using the normalised read counts obtained from DESeq2 for the multiple components of the Notch signalling pathway in the transcriptomes of B16‐F0 and B16‐BL6 mouse melanoma cell lines. Gene names are coloured according to their established relationship with the Notch pathway. On the left is indicated if a gene passed the thresholds of expression fold change (> 4), statistical significance of the differential expression, as calculated by DESeq2 (*P*‐adj < 0.01) and FDR threshold (FDR < 0.1) of the human orthologue in the human survival analysis.

To explore the role of LNFG in metastasis further, we next used our cell line transcriptome sequence data to examine the expression of Notch pathway components (Fig. 5). We observed significant changes in Notch pathway elements in B16‐BL6 such as upregulation of *Notch2, Jag1, Hes1, Esr1* and *Rbpj*, as well as downregulation of *Rfng* and *Dll3*, when compared to the B16‐F0 line. This result is in keeping with a functional role for *Lfng* expression in the metastatic phenotypes observed.

## 4 Discussion

In this study, we combined expression data from two cohorts of melanoma patients with the analysis of a selection of mouse cell lines with different metastatic capabilities to identify *LFNG* as a regulator of metastasis. We validated these results using CRISPR genome editing to transform a weakly metastatic mouse cell line to a highly metastatic line by the disruption of *Lfng*. Although our focus here was *LFNG,* many of the other genes we discovered in our analysis may also regulate melanoma metastasis. For example, the DEXD/H box helicase 60 (*DDX60*) gene, a known regulator of the antiviral response and a DNA‐/RNA‐binding protein, has been reported to be associated with the development and prognosis of squamous cell carcinoma (Fu *et al*., 2016). Similarly, *PTK2B* has been reported to be involved in the CCR7‐mediated regulation of metastasis in squamous cell carcinoma (Yue *et al*., 2015) and was found to be downregulated in our study.

Notch signal transduction occurs when a Notch ligand (Jag or delta‐like) from a sender cell binds to a Notch receptor on an adjacent receiver cell (Bray, 2016). This event triggers the proteolytic cleavage of the Notch intracellular domain (NICD). Subsequently, NICD migrates to the nucleus and binds RBP‐JK to transcribe target genes. Notch receptor–delta‐like ligand binding affinity is regulated by the fringe glycosylating enzymes (LFNG, MFNG and RFNG) (Kakuda and Haltiwanger, 2017). In this study, we show that low RNA levels of *LFNG* predict worse outcome in patients with melanoma. Further, we find that *Lfng* is downregulated in B16‐BL6 cells when compared to parental B16‐F0 cells. *Lfng* ablation in B16‐F0 cells using CRISPR enhanced the number of pulmonary metastases in support of its role in regulating metastasis. Previous work in pancreatic cancer has showed that deletion of *Lfng* in a *Kras* ^*LSL‐G12D*^ mouse model upregulates *Notch3* and *Hes1,* accelerating cell proliferation (Zhang *et al*., 2016). A role for LFNG in breast and prostate cancer has also been reported (Xu *et al*., 2012; Zhang *et al*., 2014a). In highly metastatic B16‐BL6 cells, we observed overexpression of the notch effectors, *Hes1* and *Notch2* (Fig. 5) as well as *Rbpj, Jag1, Maml2* and *Mitf*, relative to B16‐F0 cells. Notably, *delta‐like ligand* (*Dll3*), as well as the fringe genes, *Lfng* and *Rfng*, were underexpressed (Fig. 5) in comparison with the Jag ligands, *Jag1* and *Jag2*. This suggests that B16 cells are more suited to activate the Notch signalling pathway via Jag ligands.

In summary, this study shows how a cross‐species approach provides a useful framework for the identification of clinically relevant genes that play a role in metastasis. Identifying genetic markers such as *LNFG* is important as it helps to identify those patients at greatest risk of disease spread and may help guide their management.

## Author contributions

MDCV‐H, LvdW and DJA devised the experiments. MDCV‐H performed the analysis of the mouse cell line data, the comparisons with the human survival data, designed the plasmids and gRNAs, carried out the double gRNA targeting experiments and validation of all the targeting experiments on the mouse cell lines. LvdW performed all the *in vivo* experimental metastasis assays and the single gRNA targeting of the mouse cells. JN performed the QC and survival analysis on the human patient cohorts. MKS and AOS performed the RT‐qPCRs on the targeted cell lines. JN‐B and DTB provided the data for the Leeds cohort. GJ provided the data for the Lund cohort. MDCV‐H, LvdW and DJA led the project. MDCV‐H, LvdW and DJA wrote the manuscript with contributions from all authors.

## Supporting information


**Fig. S1.** Spectral Karyotyping of the B16 cell lines. Spectral karyotype analysis of ten different metaphases from (A) B16‐F0, (B) B16‐F10 and (C) B16‐BL6 cells. High levels of polyploidy, multiple chromosomal aberrations and at least one event of whole genome amplification can be observed.

**Fig. S2.** Spectral Karyotyping of the K1735 cell lines. Spectral karyotype analysis of ten different metaphases from (A) K1735‐P and (B) K1735‐M2. High levels of polyploidy, multiple chromosomal aberrations and at least one event of whole genome amplification can be observed.

**Fig. S3.** Cell line somatic variant calling and filtering strategy. Diagram showing the multiple steps followed to call single nucleotide variants and short indels from whole genome data of the murine lines in the absence of a matched normal sample from the same mouse.

**Fig. S4.** Somatic variants in murine melanoma cell lines (A) Total number of SNV and indel variants identified in each cell line. (B) Mean number SNVs identified in each mouse melanoma cell line genome. (C) Bar plot showing the mutational spectra of base substitutions identified in the lines according to the 96‐substitution type and genomic context classification.

**Fig. S5.** Variation in highly metastatic mouse cell lines**.** (A) Circos plot showing from the innermost track; somatic short indels and SNVs identified uniquely in the B16‐BL6 cell line genome, the CNVs identified in the B16‐BL6 cell line against the B16‐F0 genome, and the CNVs identified in the B16‐F0. (B) Circos plot showing from the innermost track somatic short indels, SNVs identified uniquely in the K1735‐M2 cell line genome, the CNVs identified in the K1735‐M2 cell line against the K1735‐P and the CNVs identified in the K1735‐P parental line against the C3H/HeN genome.

**Fig. S6.** Orthogonal validation of SNVs identified in the murine melanoma lines. A total of 262 variants were tested; 146 from the B16 cell line group and 116 from the K1735 lines; using three biological replicates per cell line. (A) Bar plot showing the proportion of SNVs that were validated using the Sequenom technology across three different replicates per cell line. (B) Box and whisker plot showing the proportion of validated SNVs per cell line across the three replicates, whiskers represent the upper and lower quartiles and solid thick line represents the mean.

**Fig. S7.** *Cdkn2a* genomic deletions. (A) Screenshot from the integrated genomics viewer showing the coverage of the *Cdkn2a* locus, from top to bottom, on the C57BL/6 genome data from (Keane *et al*., 2011), the B16‐F0, B16‐F10 and B16‐BL6 cell line genomes. (B) Screenshot from the integrated genomics viewer showing the coverage of the *Cdkn2a* locus, from top to bottom, on the C3H/HeJ genome data from (Keane *et al*., 2011), the K1735‐P and K1735‐M2 cell line genomes.

**Fig. S8.** Hierarchical clustering of the murine cell line RNA‐seq data. Heat map showing the hierarchical clustering of different biological replicates sequenced based on the Pearson correlation coefficient obtained from all log2(TPM + 1) values across all the protein coding genes. The two groups of cell lines can be clearly observed.

**Fig. S9.** Analysis of differentially expressed genes. Venn diagram showing the (A) overexpressed and (B) under‐expressed genes selected for qPCR validation. (C‐F) Gene expression levels with ΔΔCT value being relative to the respective parental line.

**Fig. S10.** Summary of genes assessed to identify regulators of metastatic colonisation by comparative genomics. Flow chart showing the number of genes obtained throughout the different stages of our analysis to identify regulators of metastatic colonisation in melanoma.

**Fig. S11.** Number of overlapping and concordant genes on random simulated samples. The null distribution of overlapping genes observed across 1000 samples in a set of randomisation tests with sample sizes of (A) *n* = 1290, (B) *n* = 388. The dashed red line shows the number of genes observed in our main analysis. The probability of obtaining the same number of overlapping genes as the ones observed in the real data is shown.

**Fig. S12.** Validation of reduced *Lfng* expression in B16‐BL6 cells and plasmid constructs used to generate *Lfng*‐deficient B16‐F0 cells. (A) Fold change in expression of *Lfng* in B16‐BL6 cells against B16‐F0 cells as measured by qPCR, whiskers shows the standard error and *P*‐value was calculated using two tailed *t* test from 3 biological replicates. (B) Schematics of the different plasmids used.

**Fig. S13.** *Lfng* targeting and validation of *g2d1* clone. (A) Diagram showing the targeting location of the gRNA (Lfng_g2) used in the single targeting experiment. (B) Expression analysis of *g2d1* by quantitative RT‐PCR. Fold change in expression of *Lfng* in *g2d1* cells against control cells as measured by qPCR, whiskers shows the standard error and *P*‐value was calculated using two tailed *t* test from 3 biological replicates. This frameshift mutation, although disrupting the gene, appears to cause an upregulation of *Lfng* mRNA expression although the expression difference is not statistically significant. (C) Pairwise alignment using CLUSTALX 2.1 between mouse Lfng protein (from Transcript ENSMUST00000031555) and the resulting predicted protein in clone (*g2d1*) mutated Lfng alleles. The single base insertion at the *Lfng* locus causes a frameshift that introduces a stop codon 36 amino acids downstream of the mutation site.

**Fig. S14.** *Lfng* targeting and validation of *L1* clone. (A) Diagram showing the targeting location of the gRNAs (Lfng_g2 and Lfng_g3) used in the double targeting experiment. (B) Fold change in expression of *Lfng* in *L1* cells against control cells as measured by quantitative RT‐PCR, whiskers show the standard error and *P*‐value was calculated using two tailed *t* test from 3 biological replicates. IGV screenshot showing mapped reads from the whole exome sequencing data generated from the *Lfng* KO clone (*L1*). Forward reads are shown in blue and reverse reads are shown in pink. Mismatched bases in comparison with the reference genome are highlighted above the read. The position of the targeting sites for gRNAs (C) Lfng_g2 gRNA and (D) Lfng_g3 gRNA are highlighted with a red box.

**Table S1.** Patient cohort demographic information. Demographic information for the two patient cohorts analysed where available.

**Table S2.** Predictors of patient outcome in both melanoma patient cohorts. Gene name, hazard ratios, confidence intervals, *P*‐value and corrected *P*‐values for all the genes with a *P*‐val < 0.1 after applying the FDR correction.

**Table S3.** Survival predictors gene set functional annotation enrichment. Results of the functional annotation enrichment analysis performed with DAVID with the list of gene expression predictors of outcome.

**Table S4.** Summary of chromosomal aberrations identified by spectral karyotyping of the murine melanoma cell lines. Summary of chromosomal aberrations detected by spectral karyotyping in the different mouse melanoma cell lines.

**Table S5.** Summary of Copy Number Variants identified on the mouse melanoma cell line genomes. Copy number variants (CNV) calls identified in the cell line genomes for the parental lines (B16‐F0 and K1735‐P). Somatic CNVs are reported for the metastatic lines (B16‐F10, B16‐BL6 and K1735‐M2).

**Table S6.** Differentially Expressed genes identified across the comparisons of all the murine melanoma cell lines. Information on the 1430 genes that were differentially expressed throughout all the comparisons.

**Table S7.** Oligos and gRNA sequences.
