# Supplementary figures and images for: Comparative genomics reveals that loss of lunatic fringe (LFNG) promotes melanoma metastasis
Source: Mol Oncol. 2018 Jan 7;12(2):239–55. doi: 10.1002/1878-0261.12161 (PMC5792739; doi:10.1002/1878-0261.12161)

a)

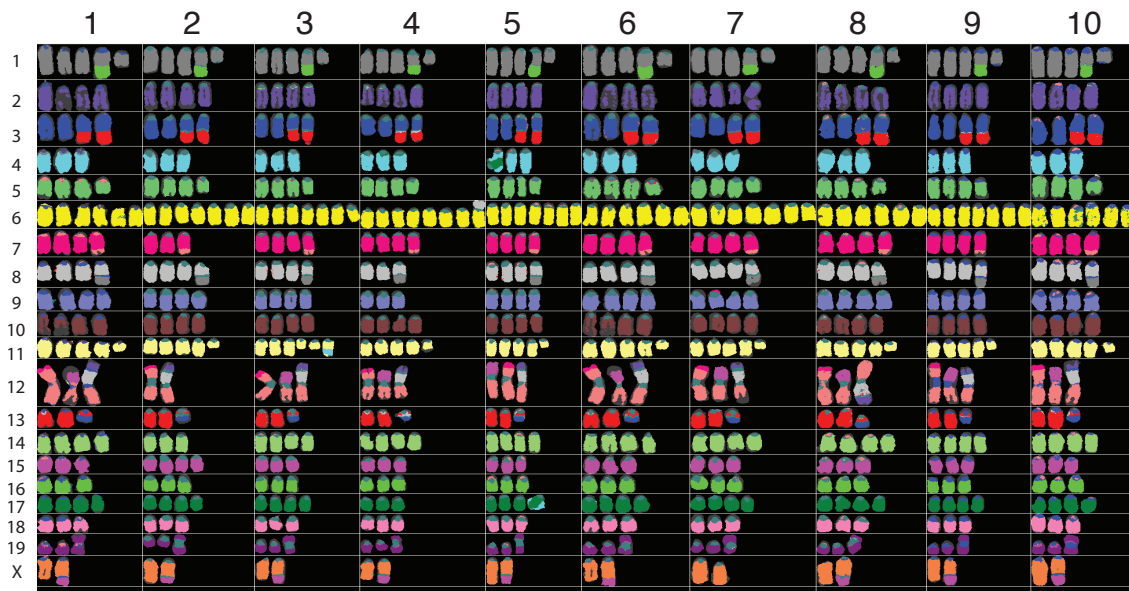

b)

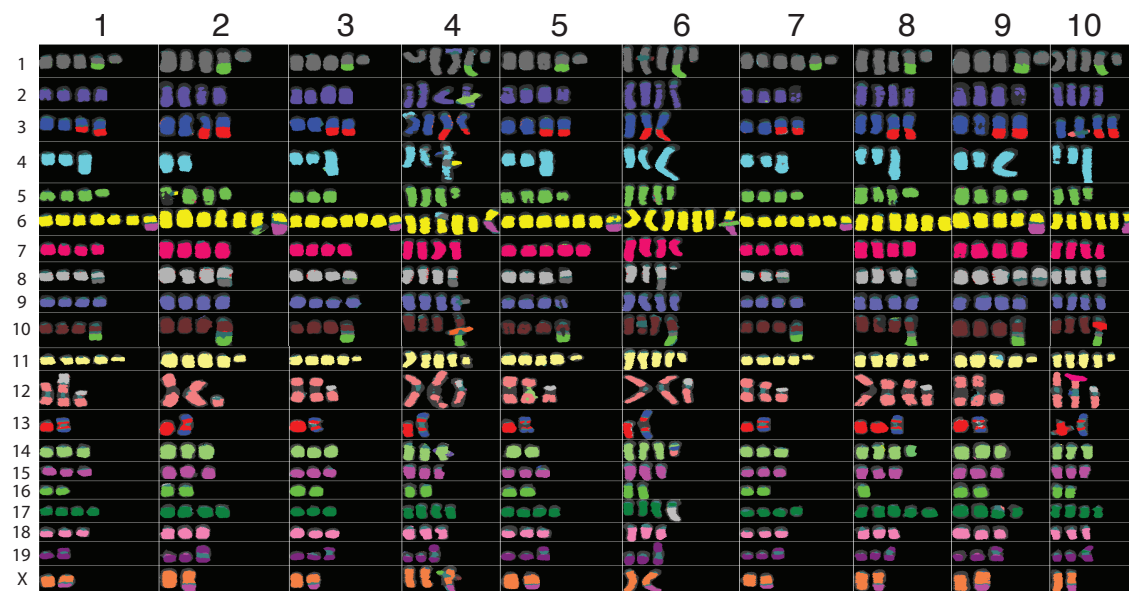

c)

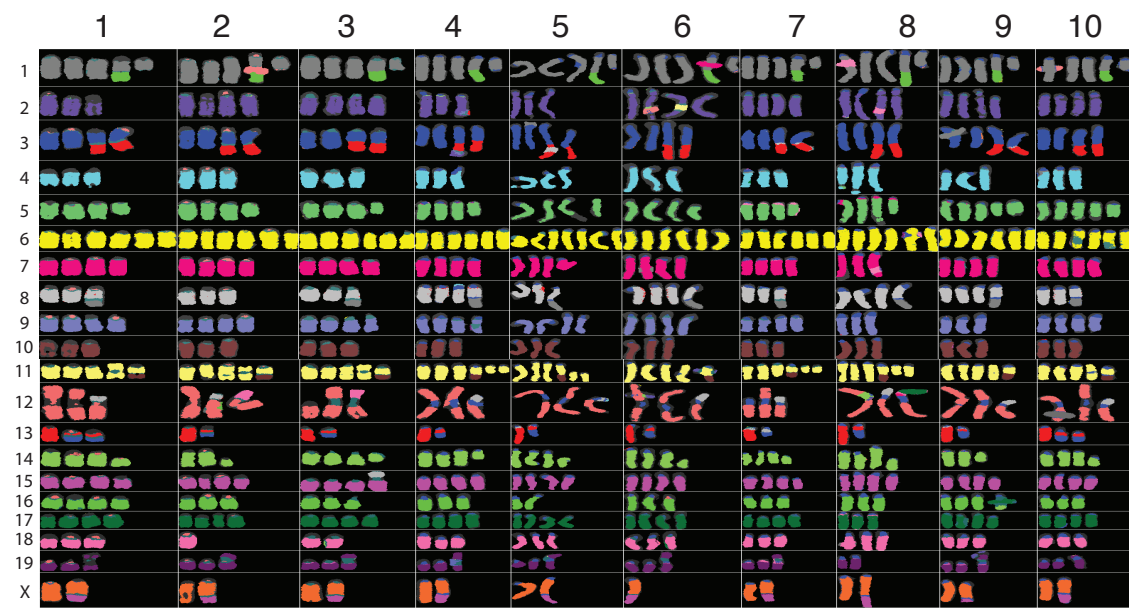

Supplement: Supplementary file 1 — Fig. S1. Spectral Karyotyping of the B16 cell lines. Spectral karyotype analysis of ten different metaphases from (A) B16‐F0, (B) B16‐F10 and (C) B16‐BL6 cells. High levels of polyploidy, multiple chromosomal aberrations and at least one event of whole genome amplification can be observed. [file MOL2-12-239-s001.pdf]

**a)**

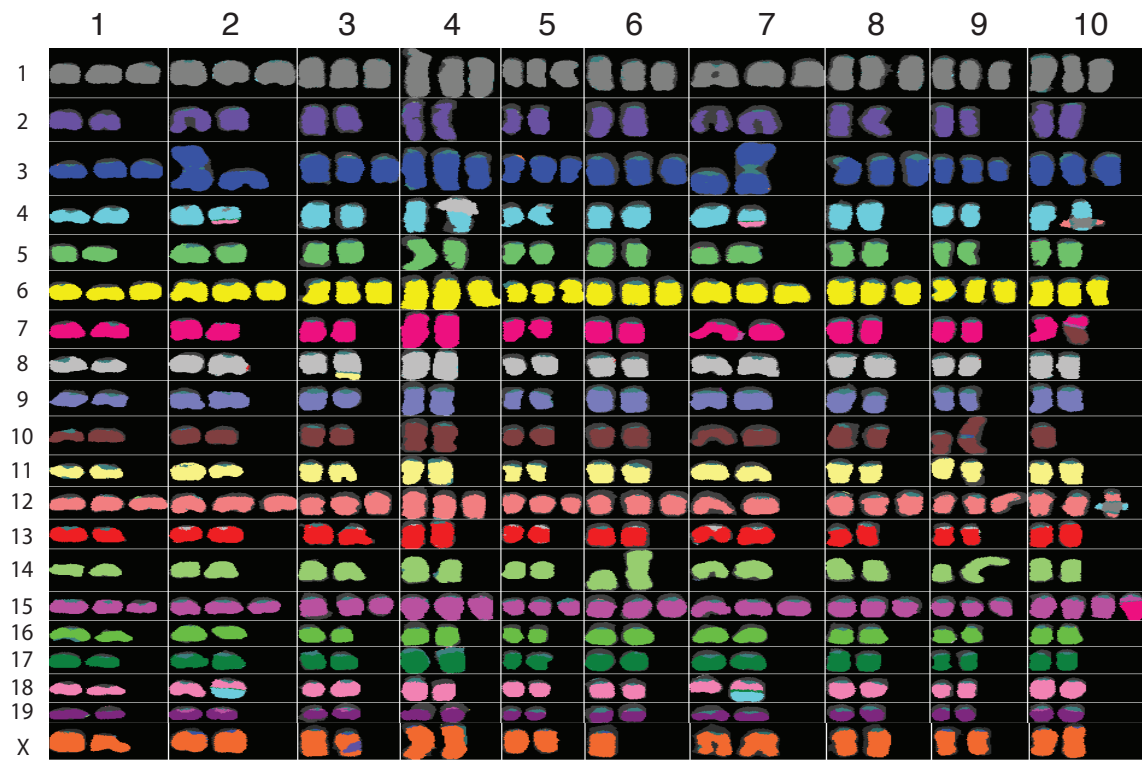

**b)**

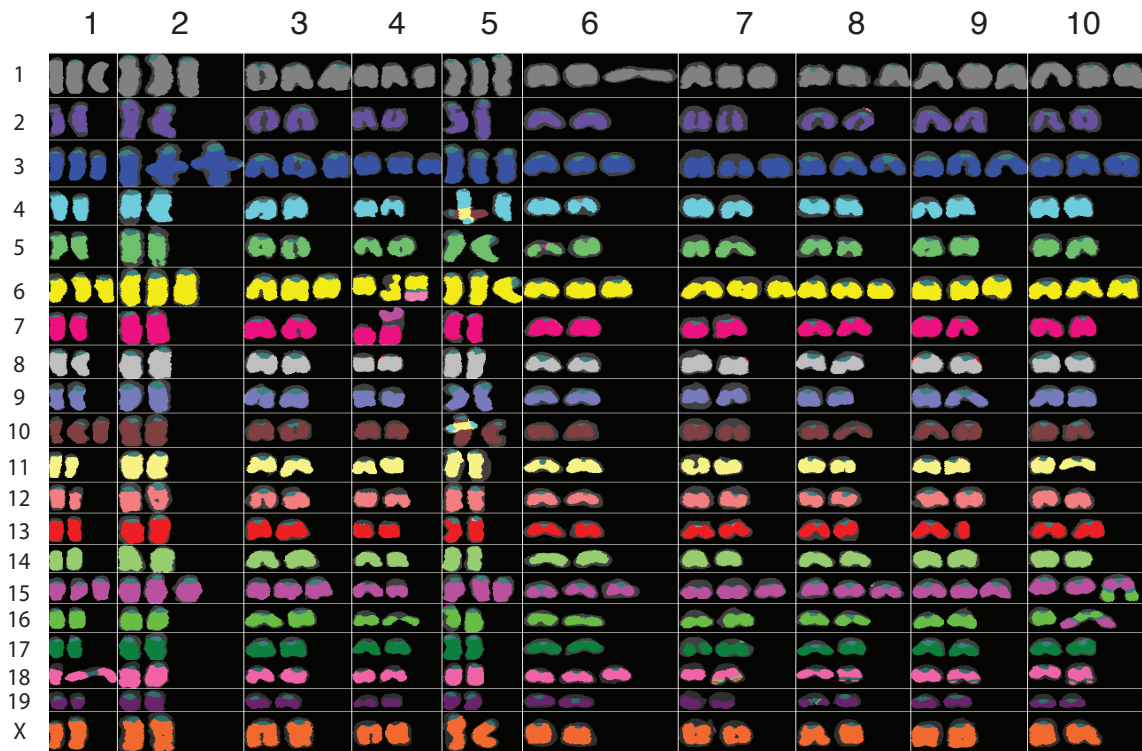

Supplement: Supplementary file 2 — Fig. S2. Spectral Karyotyping of the K1735 cell lines. Spectral karyotype analysis of ten different metaphases from (A) K1735‐P and (B) K1735‐M2. High levels of polyploidy, multiple chromosomal aberrations and at least one event of whole genome amplification can be observed. [file MOL2-12-239-s002.pdf]

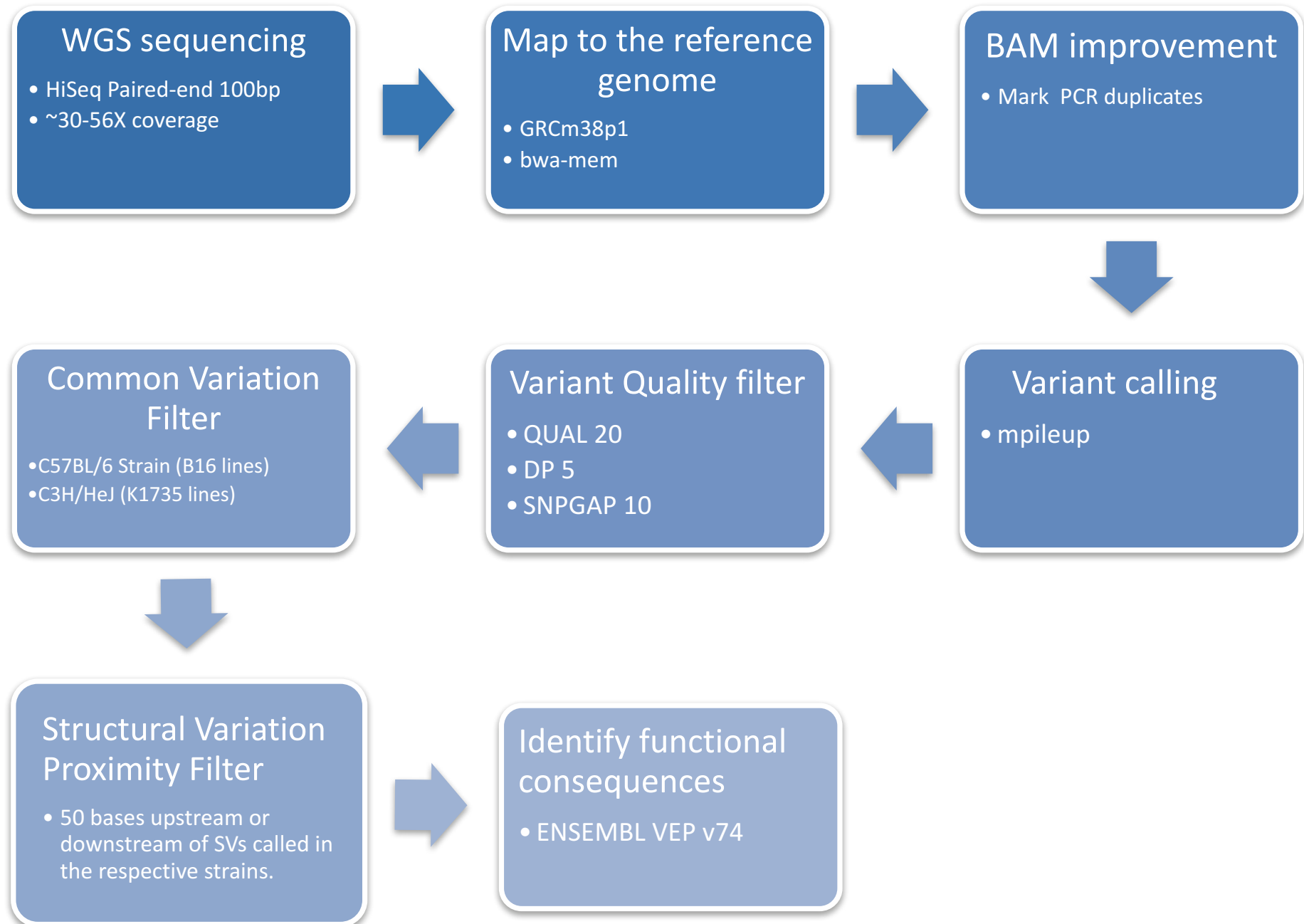

Supplement: Supplementary file 3 — Fig. S3. Cell line somatic variant calling and filtering strategy. Diagram showing the multiple steps followed to call single nucleotide variants and short indels from whole genome data of the murine lines in the absence of a matched normal sample from the same mouse. [file MOL2-12-239-s003.pdf]

**a)**

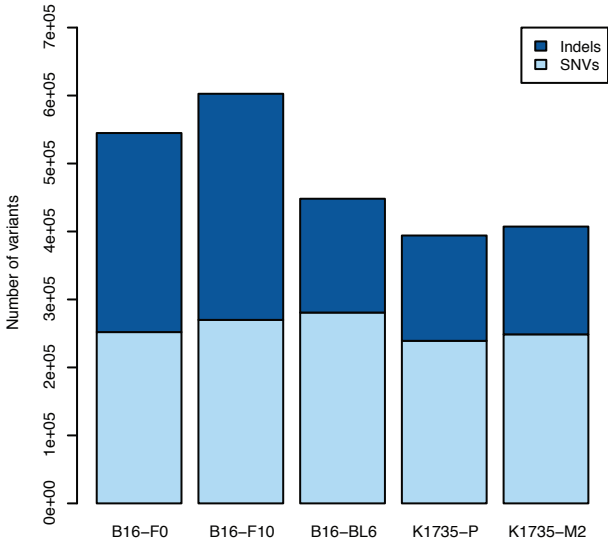

**b)**

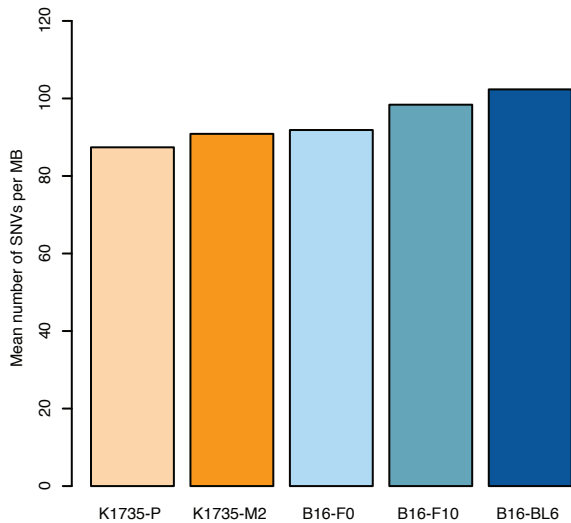

**c)**

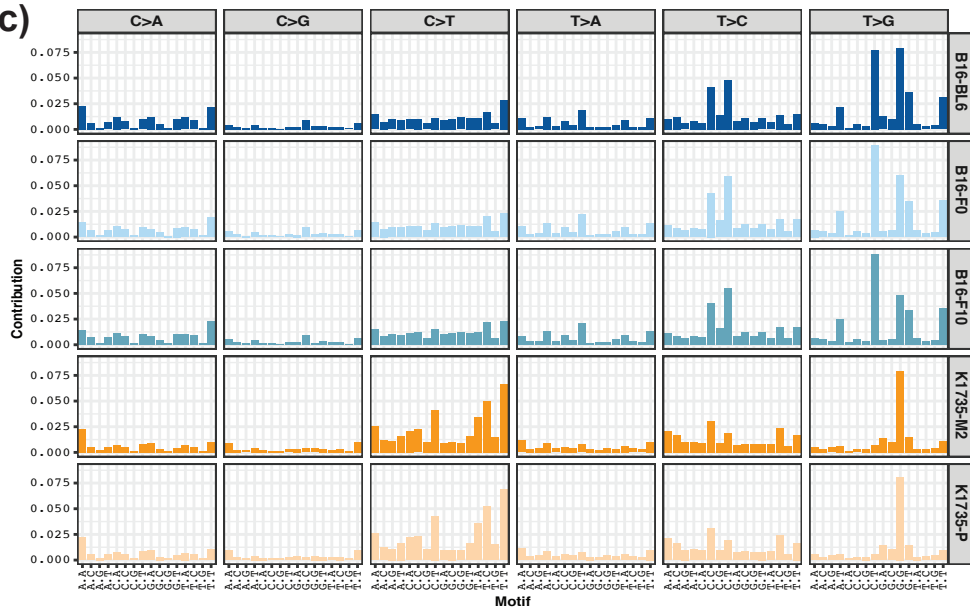

### Motif

Supplement: Supplementary file 4 — Fig. S4. Somatic variants in murine melanoma cell lines (A) Total number of SNV and indel variants identified in each cell line. (B) Mean number SNVs identified in each mouse melanoma cell line genome. (C) Bar plot showing the mutational spectra of base substitutions identified in the lines according to the 96‐substitution type and genomic context classification. [file MOL2-12-239-s004.pdf]

a)

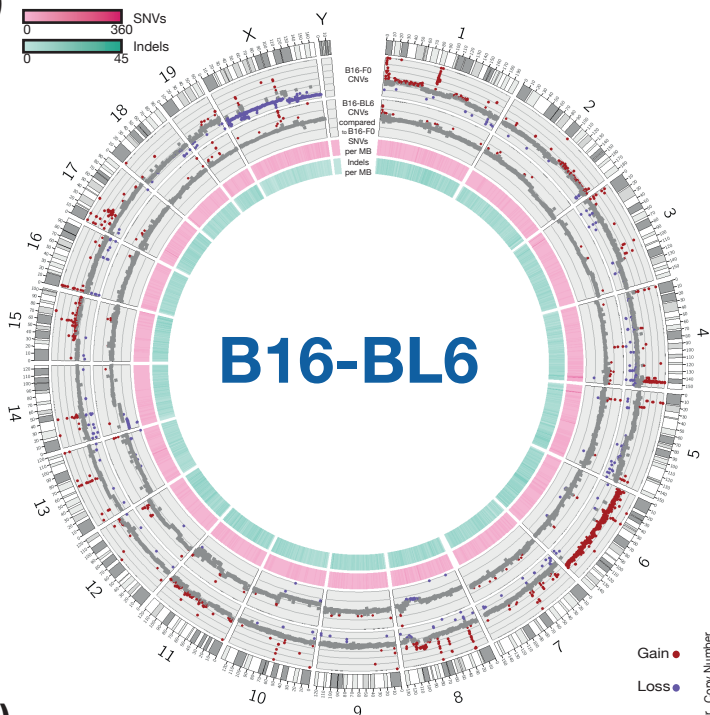

b)

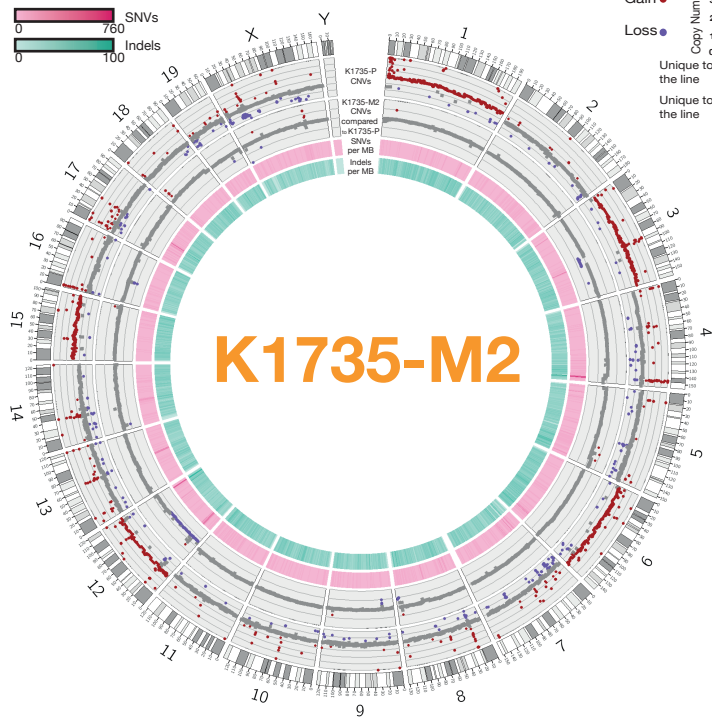

Supplement: Supplementary file 5 — Fig. S5. Variation in highly metastatic mouse cell lines. (A) Circos plot showing from the innermost track; somatic short indels and SNVs identified uniquely in the B16‐BL6 cell line genome, the CNVs identified in the B16‐BL6 cell line against the B16‐F0 genome, and the CNVs identified in the B16‐F0. (B) Circos plot showing from the innermost track somatic short indels, SNVs identified uniquely in the K1735‐M2 cell line genome, the CNVs identified in the K1735‐M2 cell line against the K1735‐P and the CNVs identified in the K1735‐P parental line against the C3H/HeN genome. [file MOL2-12-239-s005.pdf]

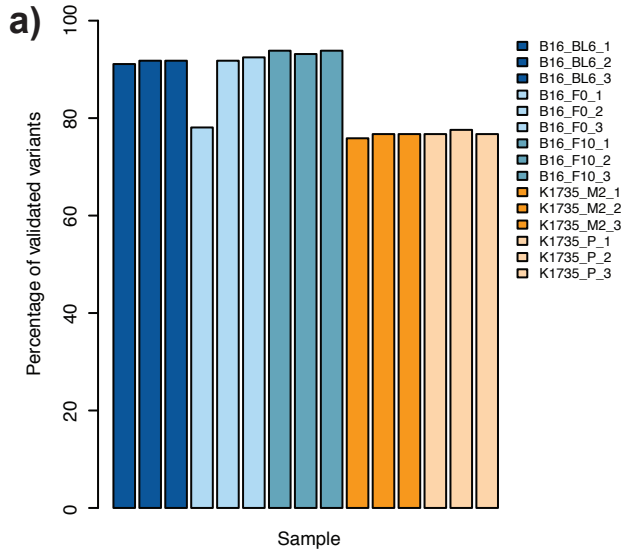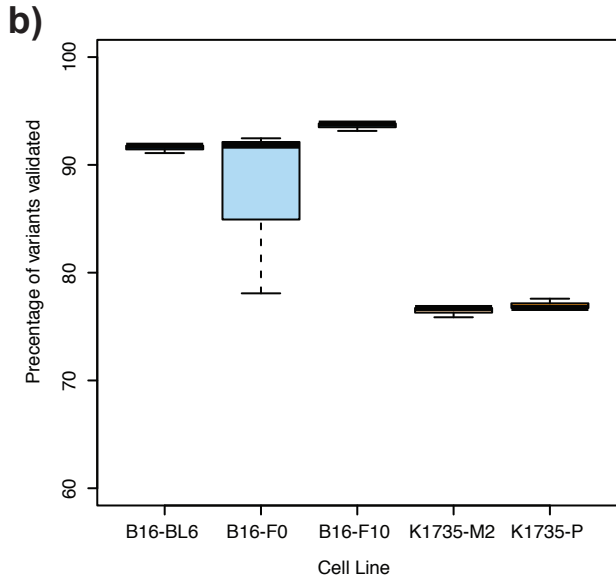

Supplement: Supplementary file 6 — Fig. S6. Orthogonal validation of SNVs identified in the murine melanoma lines. A total of 262 variants were tested; 146 from the B16 cell line group and 116 from the K1735 lines; using three biological replicates per cell line. (A) Bar plot showing the proportion of SNVs that were validated using the Sequenom technology across three different replicates per cell line. (B) Box and whisker plot showing the proportion of validated SNVs per cell line across the three replicates, whiskers represent the upper and lower quartiles and solid thick line represents the mean. [file MOL2-12-239-s006.pdf]

a)

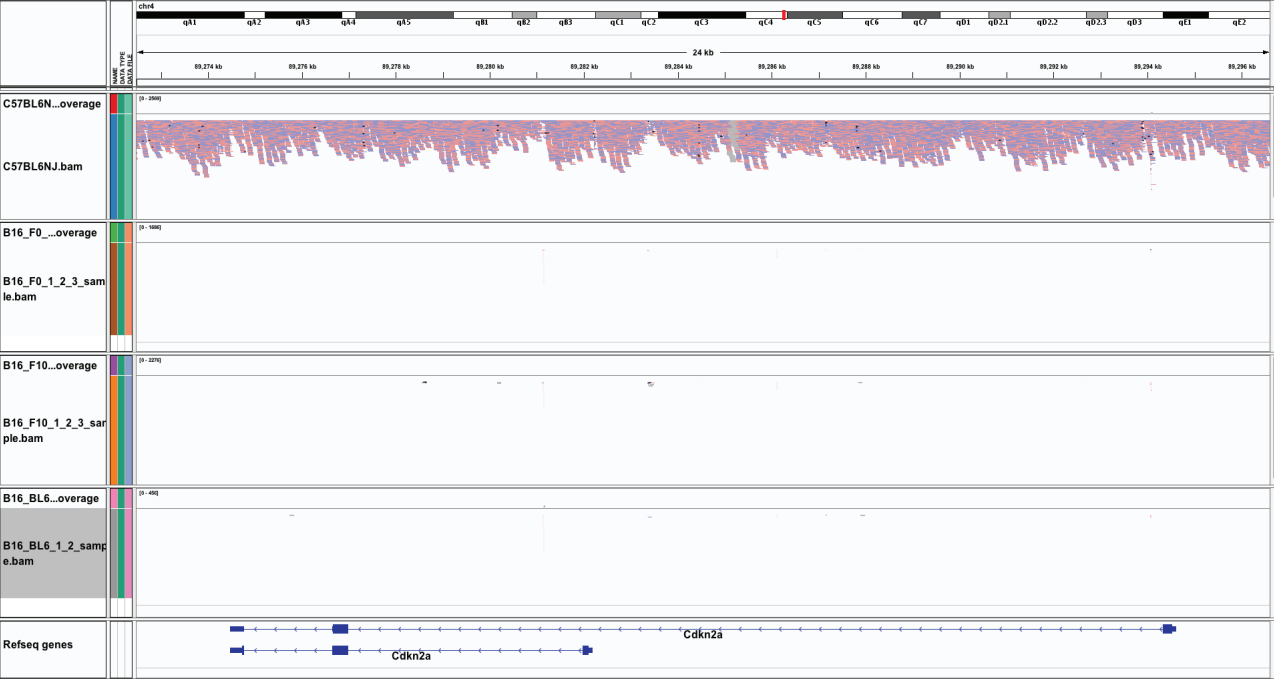

b)

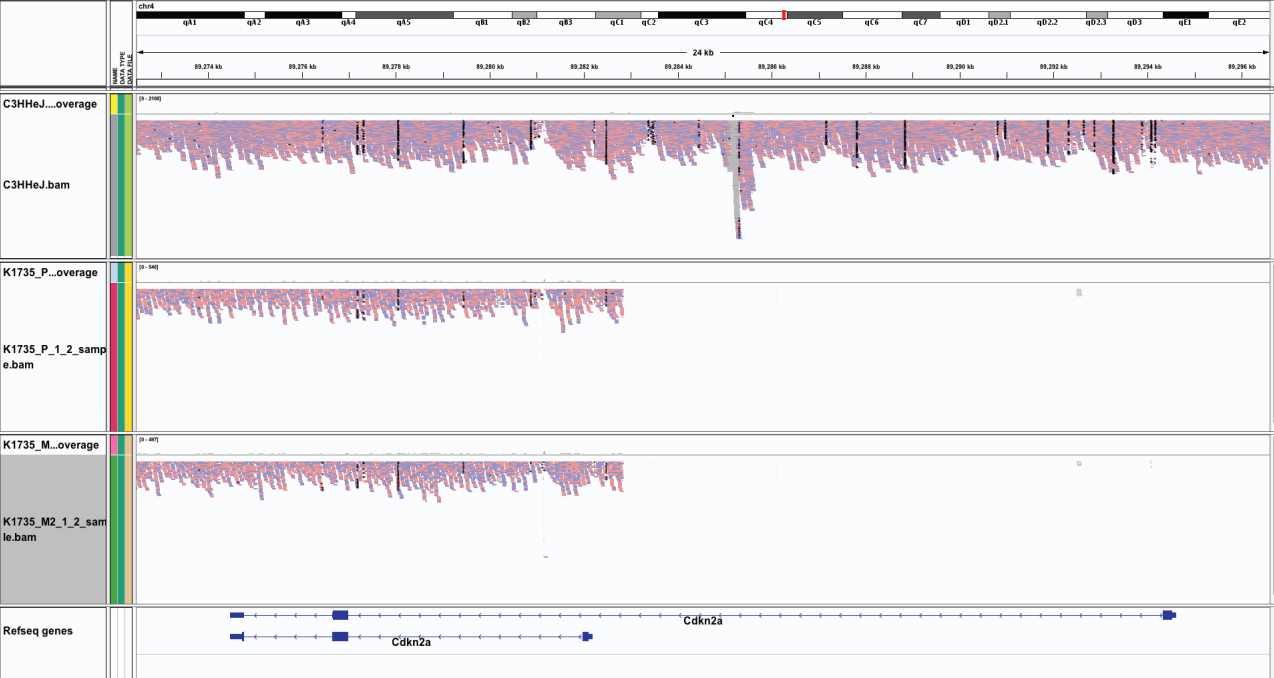

Supplement: Supplementary file 7 — Fig. S7. Cdkn2a genomic deletions. (A) Screenshot from the integrated genomics viewer showing the coverage of the Cdkn2a locus, from top to bottom, on the C57BL/6 genome data from (Keane et al., 2011), the B16‐F0, B16‐F10 and B16‐BL6 cell line genomes. (B) Screenshot from the integrated genomics viewer showing the coverage of the Cdkn2a locus, from top to bottom, on the C3H/HeJ genome data from (Keane et al., 2011), the K1735‐P and K1735‐M2 cell line genomes. [file MOL2-12-239-s007.pdf]

Colour Key  
and Histogram

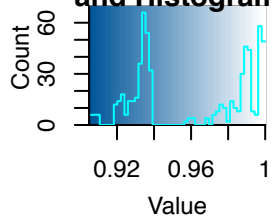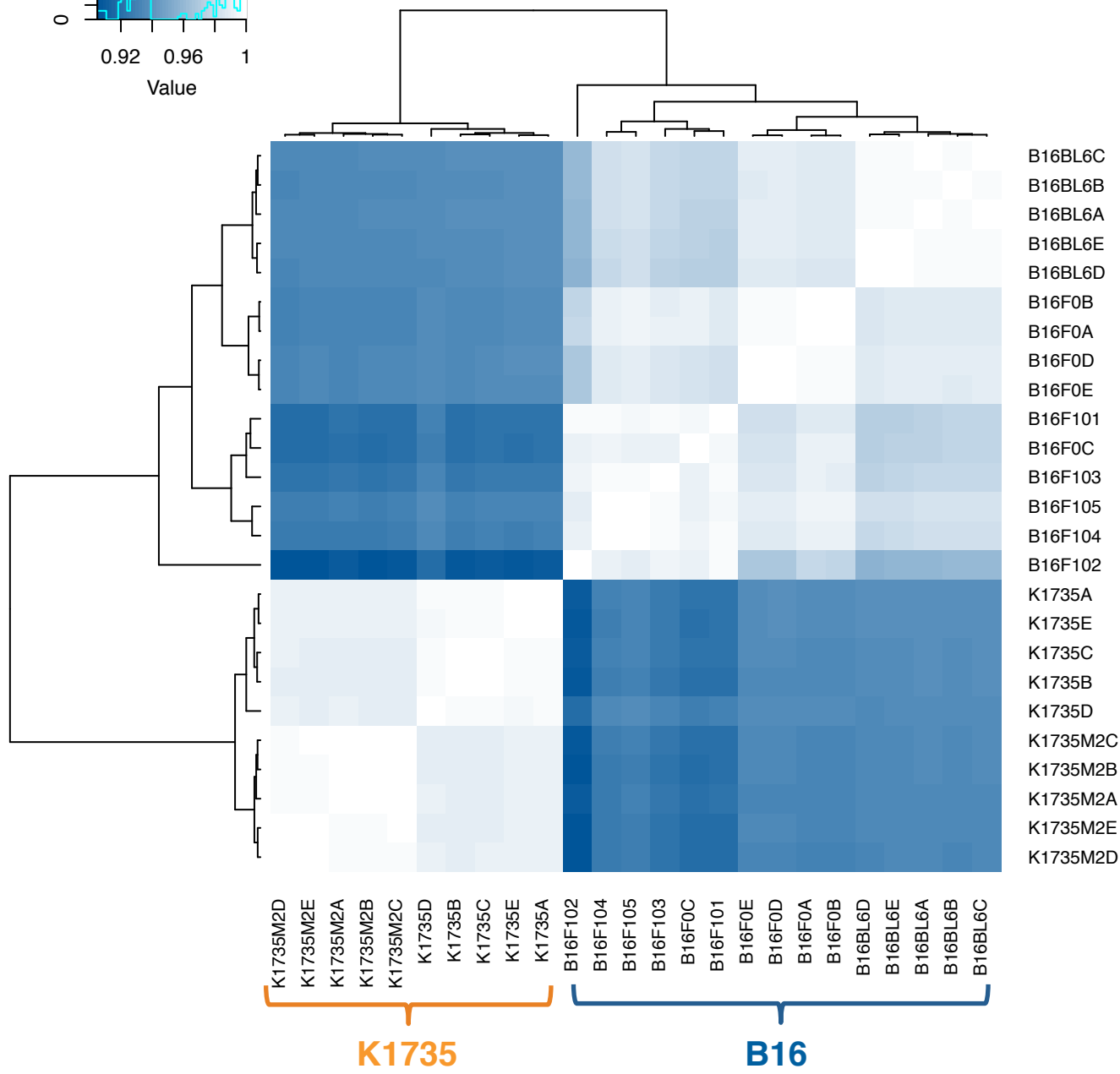

Supplement: Supplementary file 8 — Fig. S8. Hierarchical clustering of the murine cell line RNA‐seq data. Heat map showing the hierarchical clustering of different biological replicates sequenced based on the Pearson correlation coefficient obtained from all log2(TPM + 1) values across all the protein coding genes. The two groups of cell lines can be clearly observed. [file MOL2-12-239-s008.pdf]

a)

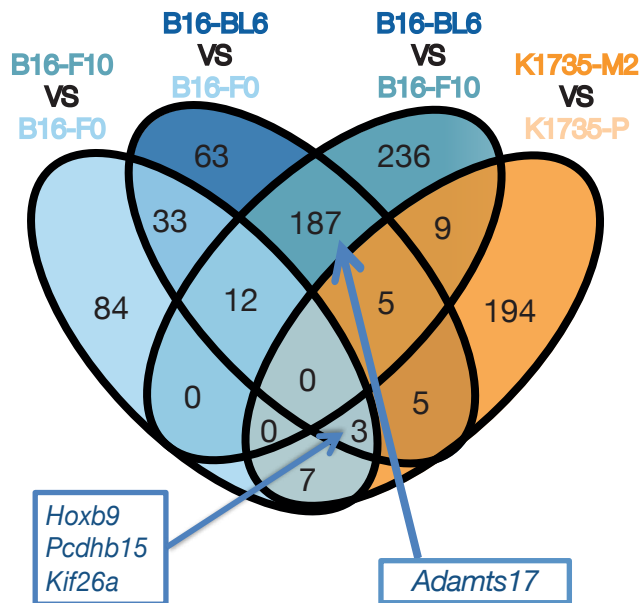

b)

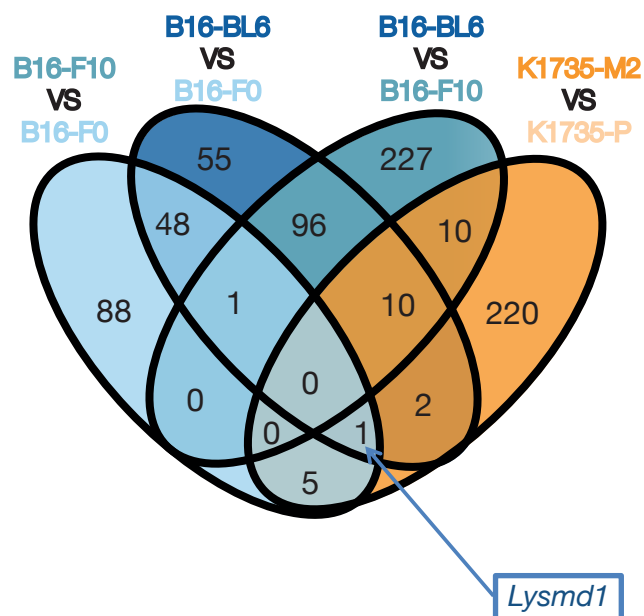

c)

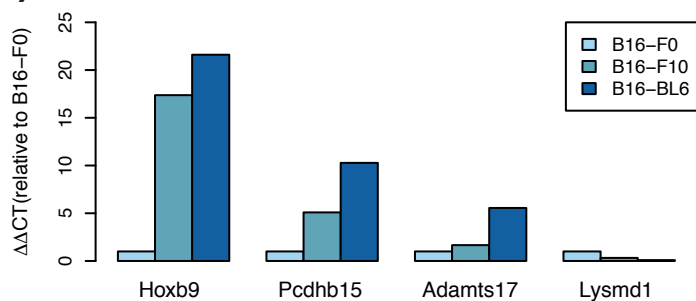

d)

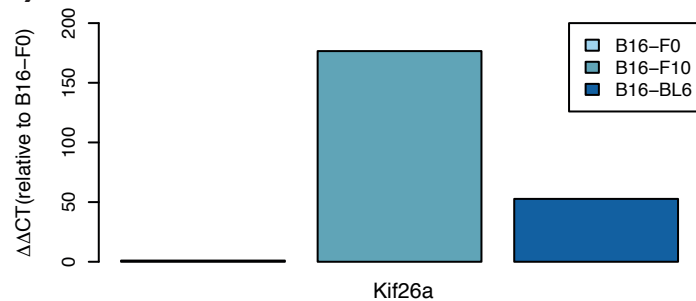

e)

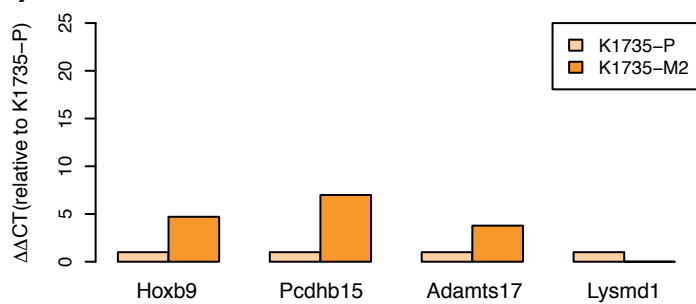

f)

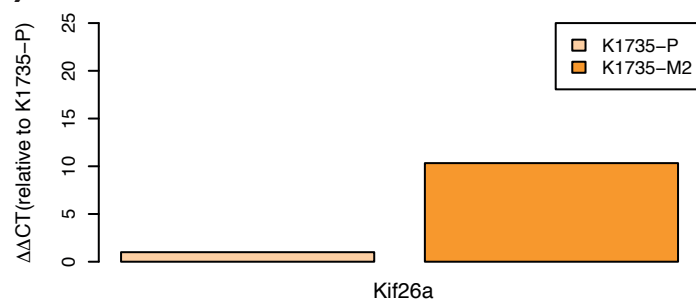

Supplement: Supplementary file 9 — Fig. S9. Analysis of differentially expressed genes. Venn diagram showing the (A) overexpressed and (B) under‐expressed genes selected for qPCR validation. (C‐F) Gene expression levels with ΔΔCT value being relative to the respective parental line. [file MOL2-12-239-s009.pdf]

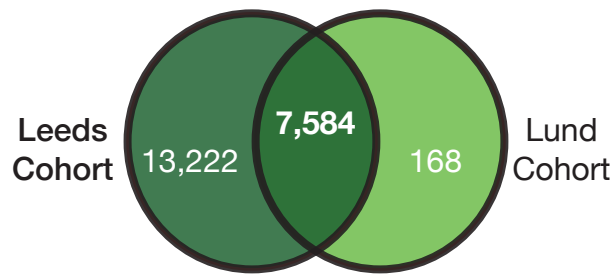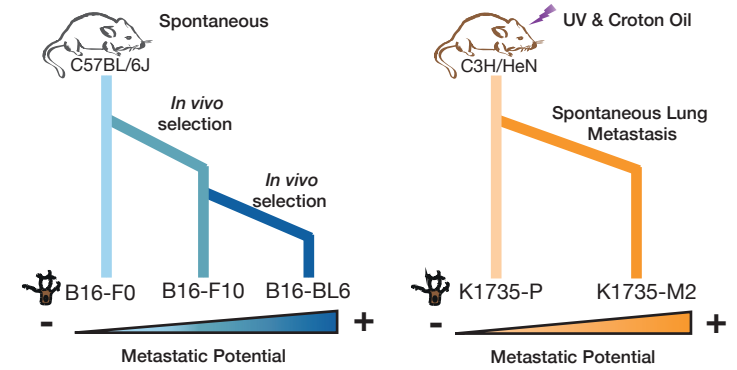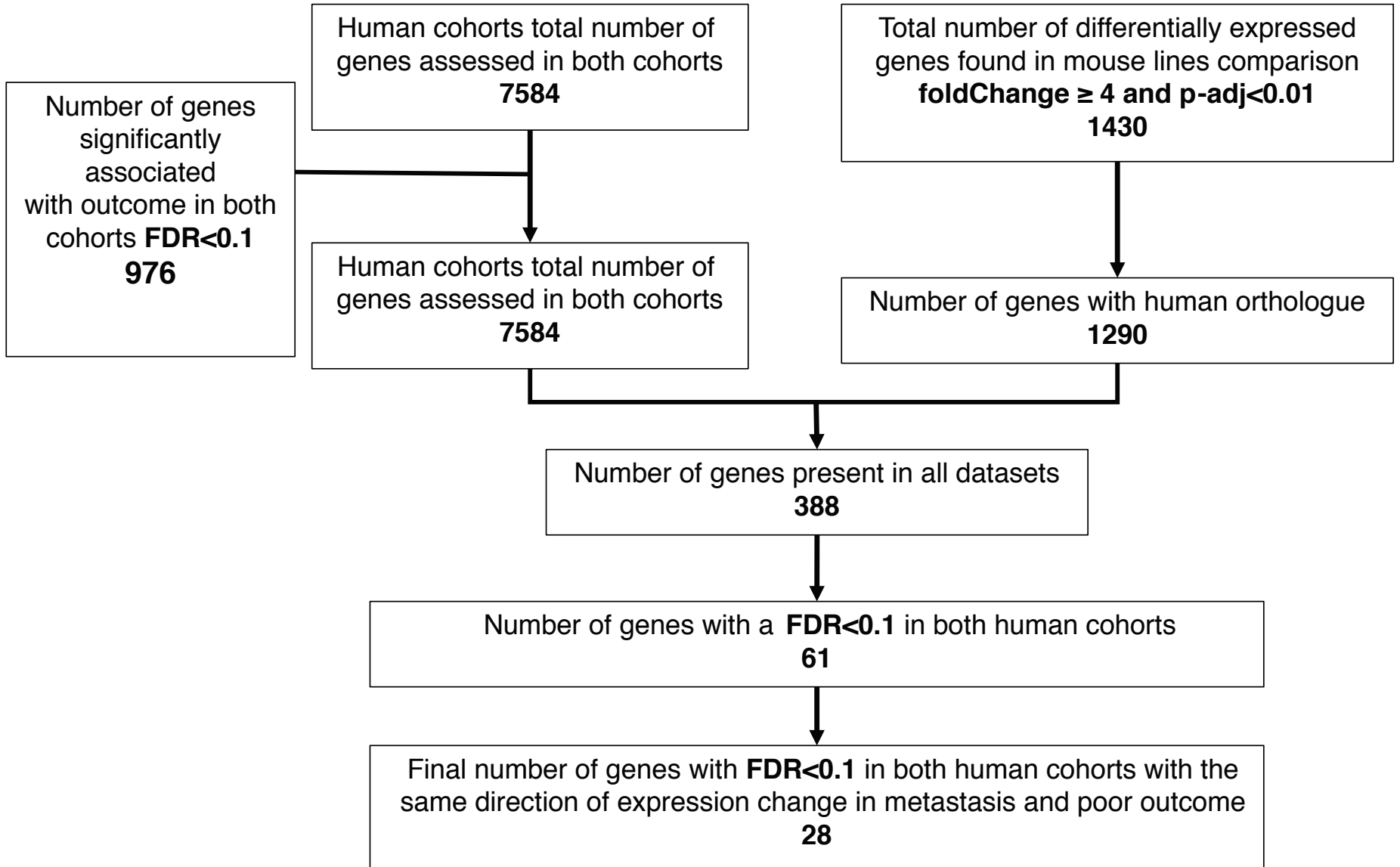

Supplement: Supplementary file 10 — Fig. S10. Summary of genes assessed to identify regulators of metastatic colonisation by comparative genomics. Flow chart showing the number of genes obtained throughout the different stages of our analysis to identify regulators of metastatic colonisation in melanoma. [file MOL2-12-239-s010.pdf]

a)

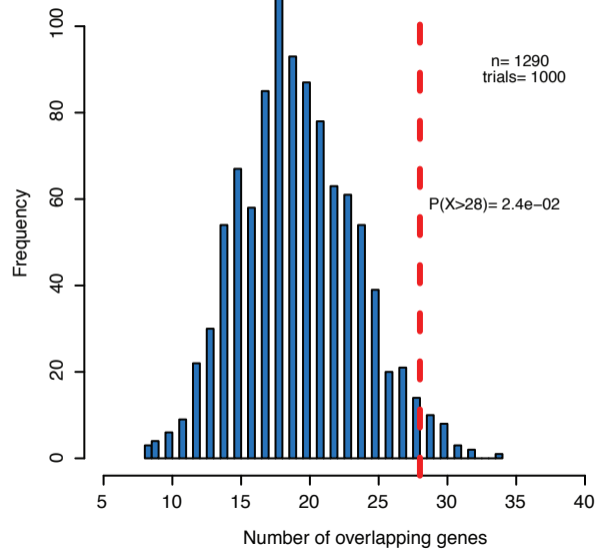

b)

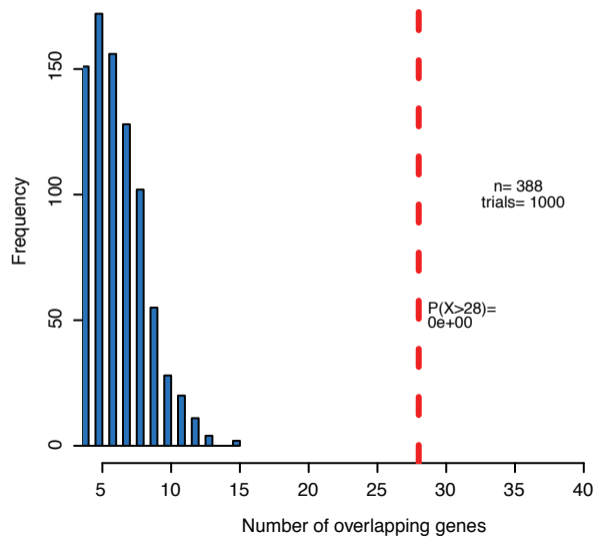

Supplement: Supplementary file 11 — Fig. S11. Number of overlapping and concordant genes on random simulated samples. The null distribution of overlapping genes observed across 1000 samples in a set of randomisation tests with sample sizes of (A) n = 1290, (B) n = 388. The dashed red line shows the number of genes observed in our main analysis. The probability of obtaining the same number of overlapping genes as the ones observed in the real data is shown. [file MOL2-12-239-s011.pdf]

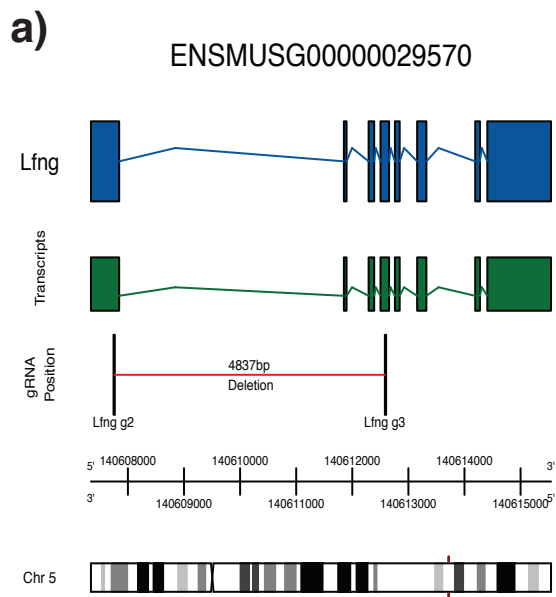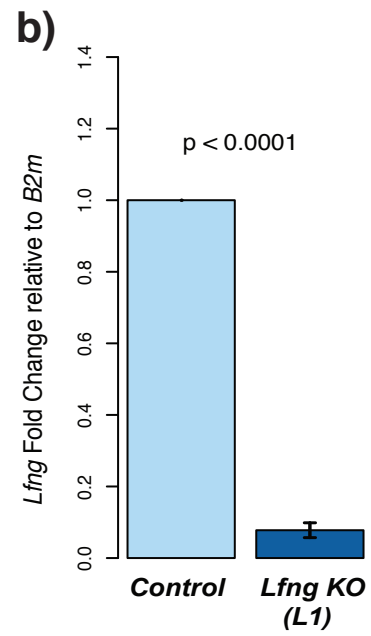

**c)**

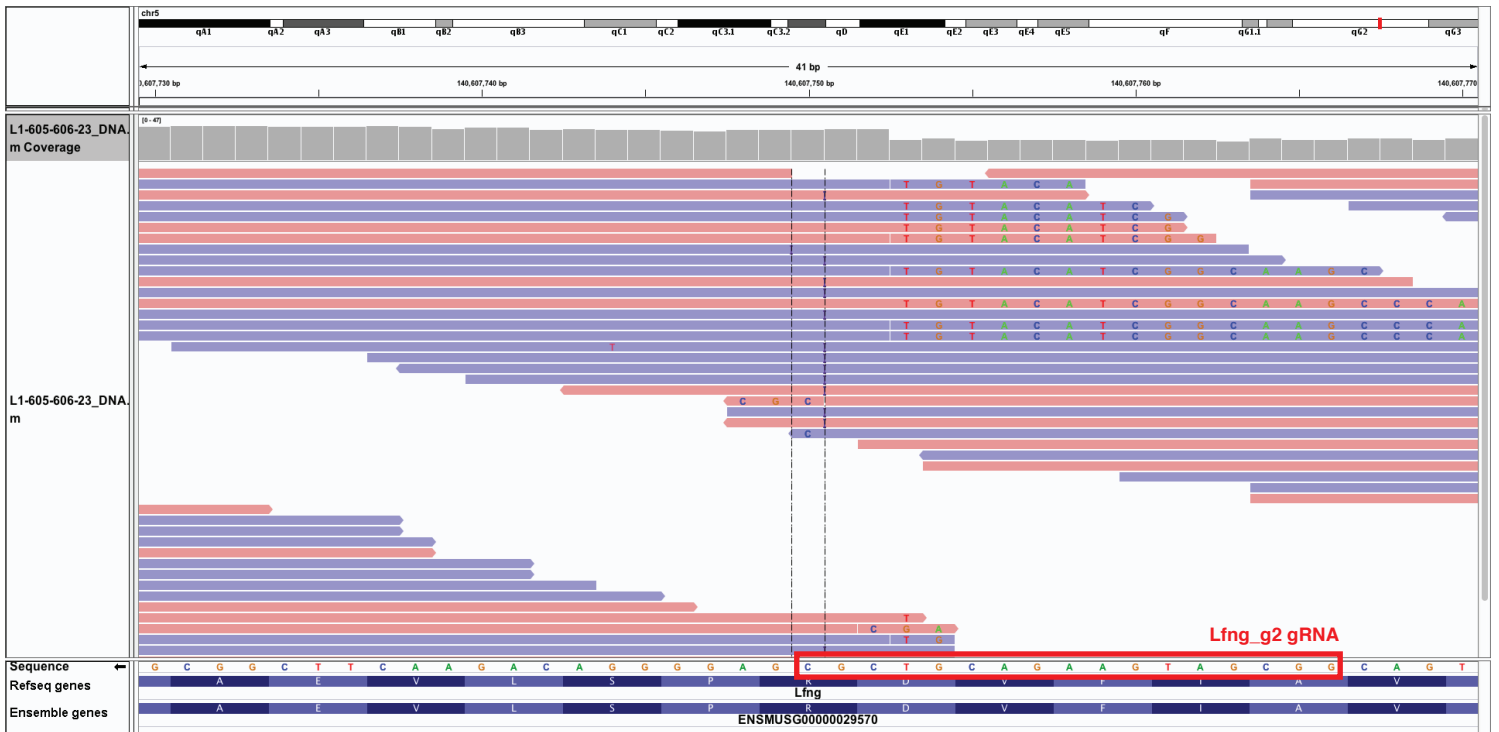

**d)**

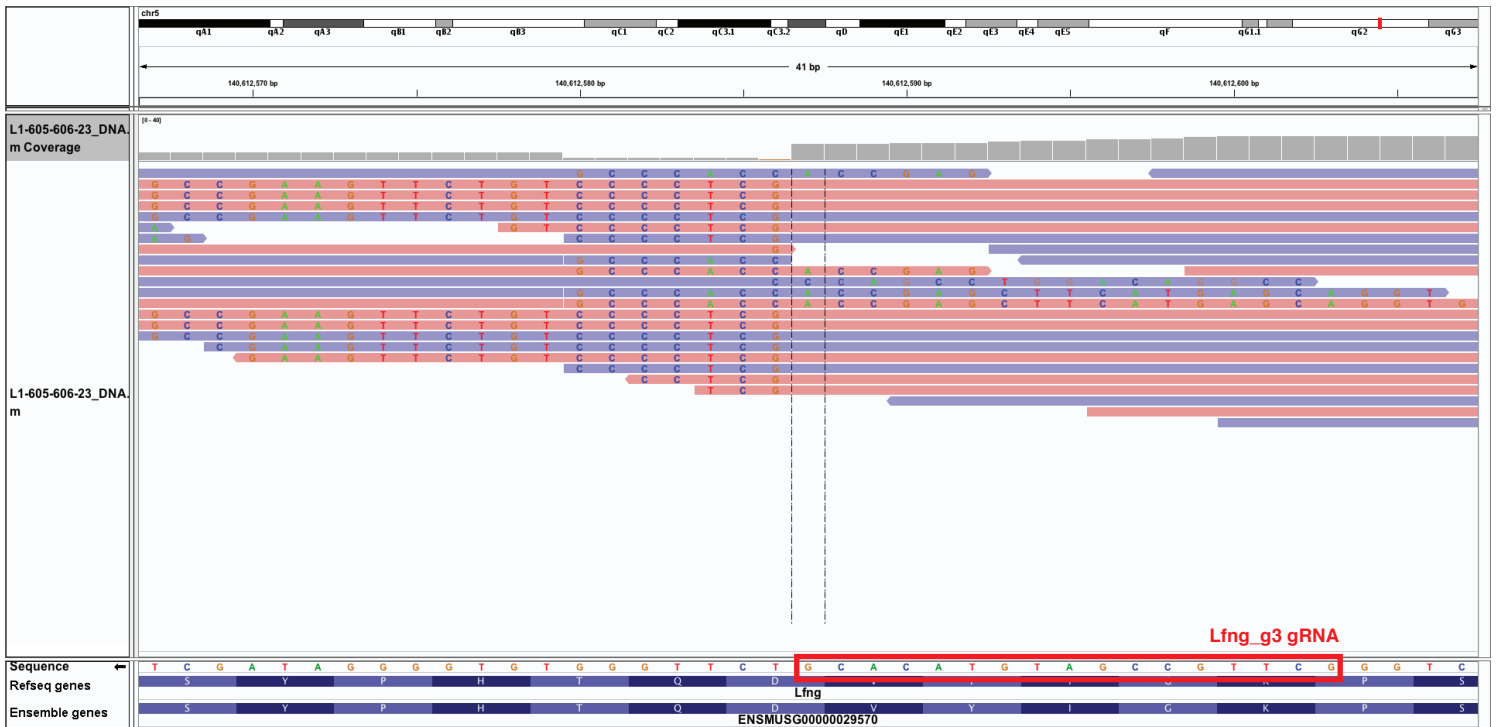

Supplement: Supplementary file 14 — Fig. S14. Lfng targeting and validation of L1 clone. (A) Diagram showing the targeting location of the gRNAs (Lfng_g2 and Lfng_g3) used in the double targeting experiment. (B) Fold change in expression of Lfng in L1 cells against control cells as measured by quantitative RT‐PCR, whiskers show the standard error and P‐value was calculated using two tailed t test from 3 biological replicates. IGV screenshot showing mapped reads from the whole exome sequencing data generated from the Lfng KO clone (L1). Forward reads are shown in blue and reverse reads are shown in pink. Mismatched bases in comparison with the reference genome are highlighted above the read. The position of the targeting sites for gRNAs (C) Lfng_g2 gRNA and (D) Lfng_g3 gRNA are highlighted with a red box. [file MOL2-12-239-s014.pdf]
