# Supplementary material for: Comparative genomics reveals that loss of lunatic fringe (LFNG) promotes melanoma metastasis
Source: Mol Oncol. 2018 Jan 7;12(2):239–55. doi: 10.1002/1878-0261.12161 (PMC5792739; doi:10.1002/1878-0261.12161)
Supplement: Supplementary file 12 — Fig. S12. Validation of reduced Lfng expression in B16‐BL6 cells and plasmid constructs used to generate Lfng‐deficient B16‐F0 cells. (A) Fold change in expression of Lfng in B16‐BL6 cells against B16‐F0 cells as measured by qPCR, whiskers shows the standard error and P‐value was calculated using two tailed t test from 3 biological replicates. (B) Schematics of the different plasmids used. [file MOL2-12-239-s012.pdf]

a)

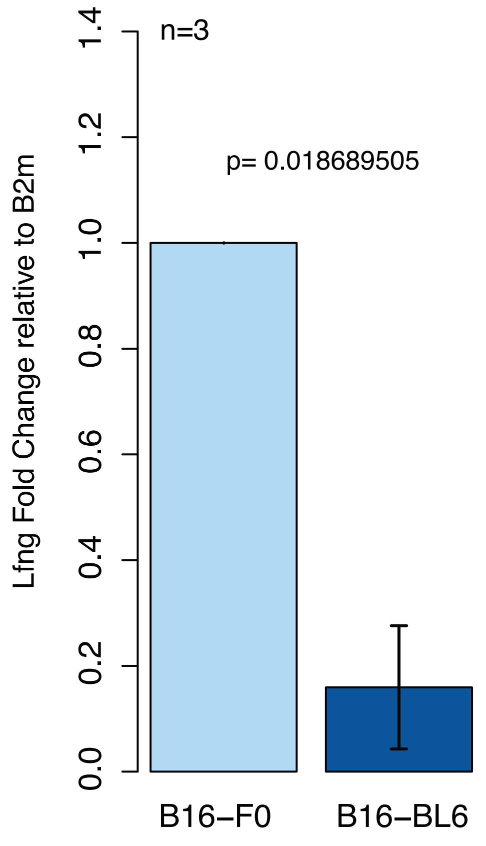
n=3
p= 0.018689505
Lfng Fold Change relative to B2m
0.0
0.2
0.4
0.6
0.8
1.0
1.2
1.4
B16–F0
B16–BL6

b)

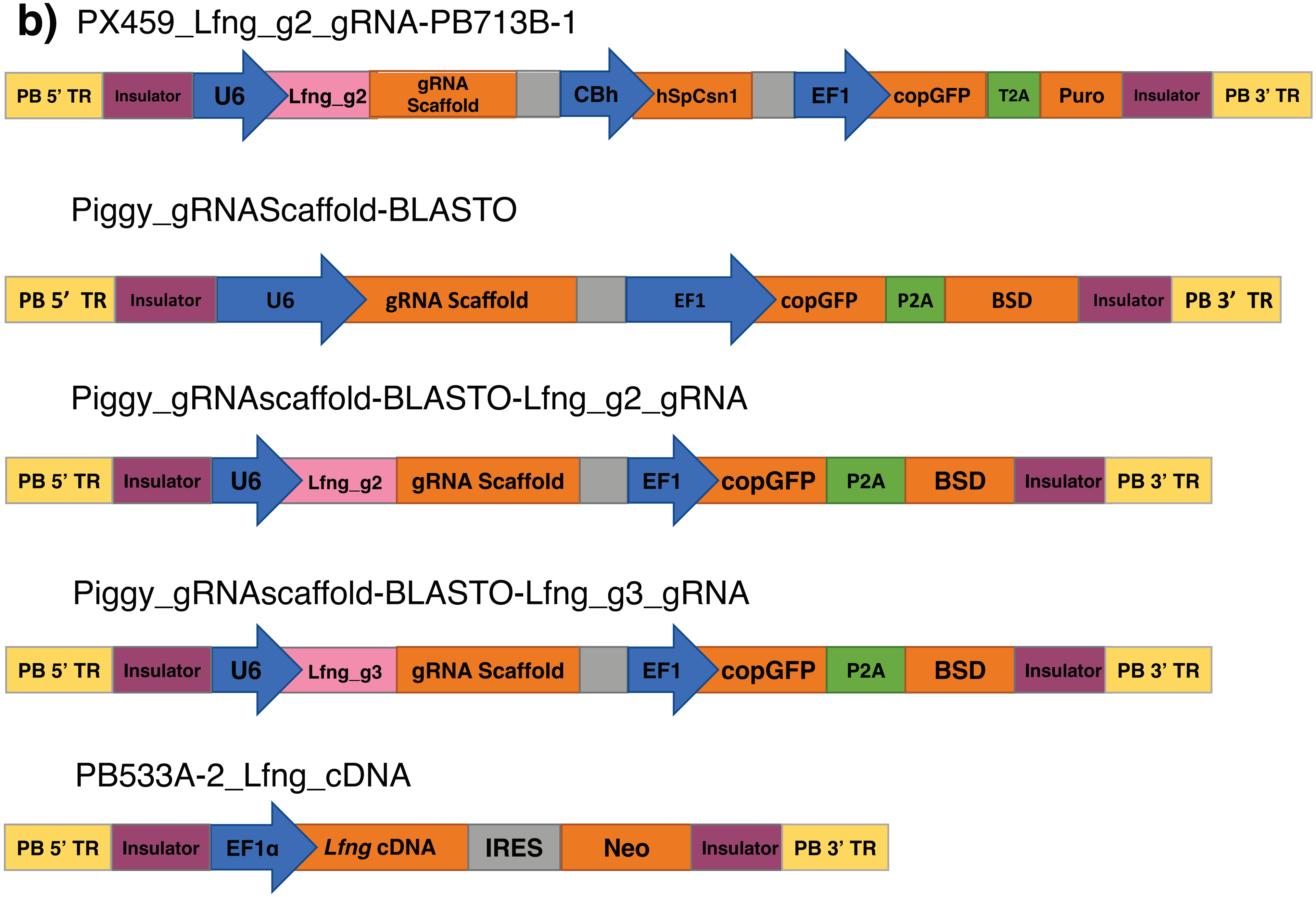
PX459_Lfng_g2_gRNA-PB713B-1
PB 5' TR
Insulator
U6
Lfng_g2
gRNA Scaffold
CBh
hSpCsn1
EF1
copGFP
T2A
Puro
Insulator
PB 3' TR
Piggy_gRNAScaffold-BLASTO
PB 5' TR
Insulator
U6
gRNA Scaffold
EF1
copGFP
P2A
BSD
Insulator
PB 3' TR
Piggy_gRNAscaffold-BLASTO-Lfng_g2_gRNA
PB 5' TR
Insulator
U6
Lfng_g2
gRNA Scaffold
EF1
copGFP
P2A
BSD
Insulator
PB 3' TR
Piggy_gRNAscaffold-BLASTO-Lfng_g3_gRNA
PB 5' TR
Insulator
U6
Lfng_g3
gRNA Scaffold
EF1
copGFP
P2A
BSD
Insulator
PB 3' TR
PB533A-2_Lfng_cDNA
PB 5' TR
Insulator
EF1α
Lfng cDNA
IRES
Neo
Insulator
PB 3' TR
