# Supplementary material for: Comparative genomics reveals that loss of lunatic fringe (LFNG) promotes melanoma metastasis
Source: Mol Oncol. 2018 Jan 7;12(2):239–55. doi: 10.1002/1878-0261.12161 (PMC5792739; doi:10.1002/1878-0261.12161)
Supplement: Supplementary file 13 — Fig. S13. Lfng targeting and validation of g2d1 clone. (A) Diagram showing the targeting location of the gRNA (Lfng_g2) used in the single targeting experiment. (B) Expression analysis of g2d1 by quantitative RT‐PCR. Fold change in expression of Lfng in g2d1 cells against control cells as measured by qPCR, whiskers shows the standard error and P‐value was calculated using two tailed t test from 3 biological replicates. This frameshift mutation, although disrupting the gene, appears to cause an upregulation of Lfng mRNA expression although the expression difference is not statistically significant. (C) Pairwise alignment using CLUSTALX 2.1 between mouse Lfng protein (from Transcript ENSMUST00000031555) and the resulting predicted protein in clone (g2d1) mutated Lfng alleles. The single base insertion at the Lfng locus causes a frameshift that introduces a stop codon 36 amino acids downstream of the mutation site. [file MOL2-12-239-s013.pdf]

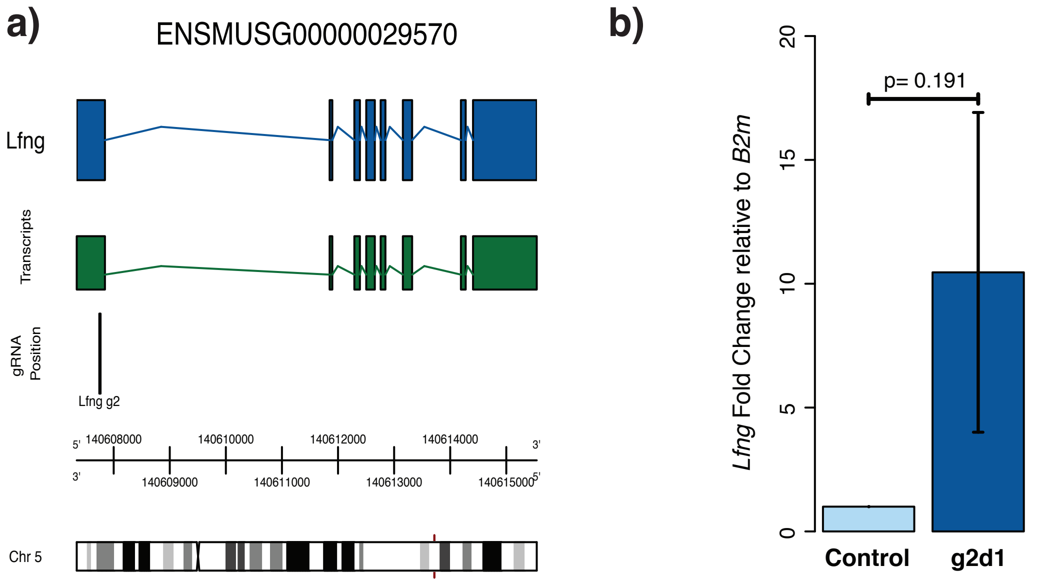

c)

```
                                  1
ENSMUST00000031555                MLQRCGRRLL LALVGALLAC LLVLTADPPP TPMPAERGRR ALRSLAGSSG
Lfng_g2_g2d1_clone_protein_Tru    MLQRCGRRLL LALVGALLAC LLVLTADPPP TPMPAERGRR ALRSLAGSSG

                                 51
ENSMUST00000031555                GAPASGSRAA VDPGVLTREV HSLSEYFSLL TRARRDADPP PGVASRQGDG
Lfng_g2_g2d1_clone_protein_Tru    GAPASGSRAA VDPGVLTREV HSLSEYFSLL TRARRDADPP PGVASRQGDG

                                101
ENSMUST00000031555                HPRPPAEVLS PRDVFIAVKT TRKFHRARLD LLFETWISRH KEMTFIFTDG
Lfng_g2_g2d1_clone_protein_Tru    HPRPPAEVLS PR-------- -RRLHRR--- ---------- ----------

                                151
ENSMUST00000031555                EDEALAKLTG NVVLTNCSSA HSRQALSCKM AVEYDRFIES GKKWFCHVDD
Lfng_g2_g2d1_clone_protein_Tru    ---------- ---------- QDHQKVSPRA A--------- ----------

                                201
ENSMUST00000031555                DNYVNLRALL RLLASYPHTQ DVYIGKPSLD RPIQATERIS EHKVRPVHFW
Lfng_g2_g2d1_clone_protein_Tru    ------RSAV RDLDLAPQGD DVHLH----- ---------- ----------

                                251
ENSMUST00000031555                FATGGAGFCI SRGLALKMGP WASGGHFMST AERIRLPDDC TIGYIVEALL
Lfng_g2_g2d1_clone_protein_Tru    ---------- ---------- ---------- ---------- ----------

                                301
ENSMUST00000031555                GVPLIRSGLF HSHLENLQQV PTTELHEQVT LSYGMFENKR NAVHIKGPFS
Lfng_g2_g2d1_clone_protein_Tru    ---------- ---------- ---------- ---------- ----------

                                351
ENSMUST00000031555                VEADPSRFRS VHCHLYPDTP WCPRSAIF
Lfng_g2_g2d1_clone_protein_Tru    ---------- ---------- --------
```
